# SelK promotes glioblastoma cell proliferation by inhibiting β-TrCP1 mediated ubiquitin-dependent degradation of CDK4

**DOI:** 10.1186/s13046-024-03157-x

**Published:** 2024-08-19

**Authors:** Jizhen Li, Lingling Zhao, Zerui Wu, Shirui Huang, Junyu Wang, Yuanyuan Chang, Li Liu, Honglei Jin, Jianglong Lu, Chuanshu Huang, Qipeng Xie, Haishan Huang, Zhipeng Su

**Affiliations:** 1 Department of Clinical Laboratory, The Second Affiliated Hospital and Yuying Children’s Hospital of Wenzhou Medical University, No.109, Xueyuan West Road, Lucheng District, Wenzhou, 325027 Zhejiang China; 2 Zhejiang Provincial Key Laboratory of Medical Genetics, Key Laboratory of Laboratory Medicine, Ministry of Education, School of Laboratory Medicine and Life Sciences, Wenzhou Medical University, Chashan, Wenzhou, 325035 Zhejiang China; 3 Department of Neurosurgery, The First Affiliated Hospital of Wenzhou Medical University, Nanbaixiang Street, Ouhai District, Wenzhou, 325000 Zhejiang China; 4 Department of Pathology, Affiliated Jinhua Hospital, Zhejiang University School of Medicine, Jinhua, 321000 Zhejiang China; 5 Department of Neurosurgery, Huashan Hospital, Shanghai Medical College, Fudan University, Shanghai, 200040 China

**Keywords:** Glioblastoma, SelK, Cell proliferation, CDK4, Endoplasmic reticulum stress

## Abstract

**Background:**

Glioblastoma (GB) is recognized as one of the most aggressive brain tumors, with a median survival of 14.6 months. However, there are still some patients whose survival time was greater than 3 years, and the biological reasons behind this clinical phenomenon arouse our research interests. By conducting proteomic analysis on tumor tissues obtained from GB patients who survived over 3 years compared to those who survived less than 1 year, we identified a significant upregulation of SelK in patients with shorter survival times. Therefore, we hypothesized that SelK may be an important indicator related to the occurrence and progression of GBM.

**Methods:**

Proteomics and immunohistochemistry from GB patients were analyzed to investigate the correlation between SelK and clinical prognosis. Cellular phenotypes were evaluated by cell cycle analysis, cell viability assays, and xenograft models. Immunoblots and co-immunoprecipitation were conducted to verify SelK-mediated ubiquitin-dependent degradation of CDK4.

**Results:**

SelK was found to be significantly upregulated in GB samples from short-term survivors (≤ 1 year) compared to those from long-term survivors (≥ 3 years), and its expression levels were negatively correlated with clinical prognosis. Knocking down of SelK expression reduced GB cell viability, induced G0/G1 phase arrest, and impaired the growth of transplanted glioma cells in nude mice. Down-regulation of SelK-induced ER stress leads to a reduction in the expression of SKP2 and an up-regulation of β-TrCP1 expression. Up-regulation of β-TrCP1, thereby accelerating the ubiquitin-dependent degradation of CDK4 and ultimately inhibiting the malignant proliferation of the GB cells.

**Conclusion:**

This study discovered a significant increase in SelK expression in GB patients with poor prognosis, revealing a negative correlation between SelK expression and patient outcomes. Further mechanistic investigations revealed that SelK enhances the proliferation of GB cells by targeting the endoplasmic reticulum stress/SKP2/β-TrCP1/CDK4 axis.

**Supplementary Information:**

The online version contains supplementary material available at 10.1186/s13046-024-03157-x.

## Introduction

Glioblastoma (GB) is one of the deadliest and most recalcitrant malignant solid tumors of the central nervous system [1, 2]. Despite advances in surgical, pharmacologic, and radiotherapeutic treatments, the prognosis for patients with GB remains extremely poor, with a median survival of 14.6 months [3–5], and survival rates at 1-, 2-, 3-, and 5-years of only 39.3%, 16.9%, 9.9%, and 5.5%, respectively [6]. The small number of GB patients who survive ≥ 3 years are known as long-term survivors (LTS) [7–9]. This group of patients remains important in investigating the determinants of the ability of current treatments to produce lasting efficacy [10–12].

Targeted therapy is emerging as a viable therapeutic option to improve outcomes in GB patients. Research has shown that the use of Bevacizumab (a VEGF inhibitor) and Dacomitinib (an EGFR inhibitor) shows promise in clinical practice [13, 14]. However, GB is a highly heterogeneous tumor, and single-target approaches have limited efficacy. Therefore, the future of GB therapy lies in identifying multiple targets and embracing multimodal combination therapies. Accordingly, there is an urgent need to explore new and highly specific therapeutic targets on the tumor cells that can enhance the sensitivity of current treatments and provide increased hope for long-term benefits for GB patients.

The malignant proliferation of tumor cells is a key factor in the negative impact on patients’ health [15]. Despite progress made in research, which has revealed numerous genetic and protein abnormalities, dysregulated signal transduction pathways and loss of cell cycle control remain prominent issues in GB [16, 17]. Several studies have demonstrated that reducing tumor tissue volume and inhibiting tumor cell proliferation can both alleviate neurological symptoms and optimize the effectiveness of chemotherapy and radiation treatments [18–20].

SelK is a member of the Selenoprotein family [21] that is widely expressed in various tissues, with particularly high levels found in four brain regions [22, 23]. Functionally, SelK is involved in calcium ion (Ca^2+^) homeostasis regulation. In neuronal cells, SelK knockout increases neuronal apoptosis and intracellular Ca^2+^ concentration in mice by activating calpain /caspase-12, leading to impaired cognitive ability [24]. In addition to its involvement in homeostasis regulation, SelK has been implicated in endoplasmic reticulum (ER) stress regulation [25] and acts as a tumor suppressor in gastric cancer and human chorionic carcinoma [26, 27]. In addition, SelK is also implicated as a key player in the biological process by which MicroRNA-181 inhibits the proliferation, drug sensitivity, and invasion of human glioma cells [28]. However, more experiments are needed to confirm this, which makes further research on its role in the development of GB essential. Such research may open new avenues for identifying therapeutic targets in gliomas, including GB.

This study sought to increase the understanding of the involvement of SelK in the advancement of GB. Specifically, it was hoped the research here would show that SelK can function as a tumor enhancer by controlling the cell cycle of GB cells through the ER Stress/SKP2/β-TrCP1/CDK4 pathway.

## Results

### SelK is up-regulated in human GB tissue associated with poor prognosis

**Fig. 1:**
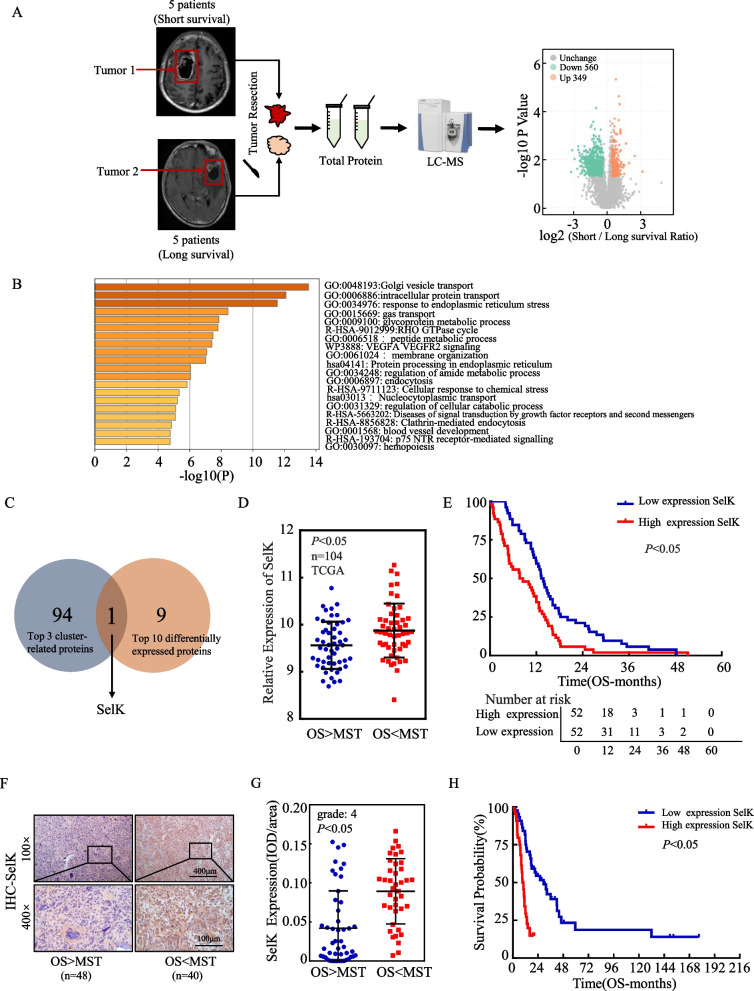
SelK is up-regulated in human GB tissue with poor prognosis. **A** Quantitative proteomic analysis of TMT-tagged, up- and down-regulated proteins demonstrated as volcano plots. **B** GO enrichment analysis of upregulated proteins. **C** Venn diagram showing the top 3 cluster-related proteins and the top 10 differentially expressed proteins by the proteomic analysis. **D** Differential mRNA expression analysis of SelK in GB tissues from TCGA database (MST is the median survival time). **E** The relationship between SelK expression and overall survival (OS) of patients was analyzed using survival curves with data from TCGA. **F** SelK protein expression in GB tissues (*n* = 88) analyzed by IHC staining. (MST is the median survival time). **G** SelK protein expression levels analyzed by calculating IOD/area. **H** The survival curves indicate that SelK expression correlates with overall survival of the patients. (*n* = 88) *Significant difference at *p* < 0.05. Data are expressed as means ± SD. Log-rank was used for survival analysis

Most GB patients have a survival period of ≤ 1 year, but a small number of patients still have a survival period of ≥ 3 years. A comprehensive analysis of the clinical data revealed a highly significant difference in the prognosis of patients who were not statistically-different in age and who were all diagnosed pathologically as GB (with wild-type isocitrate dehydrogenase [IDH]), and who were all treated with concurrent radiotherapy (Supplementary Table 1). Tissue samples from 10 patients were divided into two groups based on survival time. Five patients who survived for 3 years or more were classified into the long survival group, and five patients who survived for 1 year or less were classified into the short survival group. All samples were subjected to TMT-based quantitative proteomic analysis (Supplementary file). The results showed that in comparison to the tumor tissues of GB patients with longer survival, 349 proteins were markedly up-regulated in the tumor tissues of patients with shorter survival, and 560 genes were significantly down-regulated (Fig. 1A). Differential proteins were analyzed using GO analysis (Fig. 1B). The top three cluster-related proteins from gene enrichment analysis intersected with the top 10 differentially expressed proteins, identifying SelK as the only common protein (Fig. 1C). In the TCGA database, SelK expression was significantly higher in patients with poor prognosis compared to those with better prognosis (Fig. 1D), and overall survival (OS) was significantly lower in patients with high SelK expression (Fig. 1E).

Building on these findings, the potential role of SelK in GB development was investigated by analyzing its expression in 88 cases of GB tissues (Supplementary Table 2) via immunohistochemical analysis. The results were then analyzed in the context of patient survival rates. These analyses revealed a significant up-regulation of SelK in tissues of patients with shorter survival periods (Fig. 1F, G). In addition, a negative correlation between SelK expression and overall survival rates among GB patients (*n* = 88) was revealed (Fig. 1H). These findings suggest that SelK may serve as a crucial marker for GB prognosis and play a key role in its development.

### SelK is up-regulated in human GB cell lines and its knockdown inhibits the growth of GB cells

**Fig. 2:**
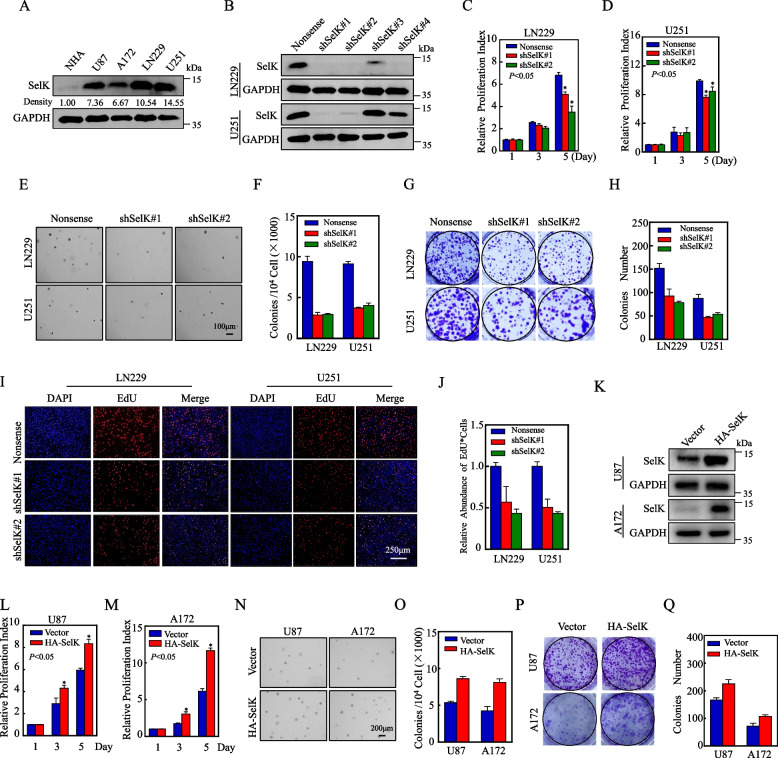
SelK is up-regulated in human GB cell lines and its knockdown inhibits the growth of GB cells. **A** SelK protein expression in normal astrocytes (NHA) and human GB cell lines (Western blot). The numbers below represent the ratio of the gray value of the SelK protein band relative to that of internal control. **B** Efficiency of SelK protein expression knockdown in LN229 and U251 stable cell lines (Western blot). **C**,** D** ATP assay to determine effect of SelK knockdown on proliferation of LN229 and U251 cells (each assay repeated three times independently). **E**, **F** Soft agar assay to determine effect of SelK knockdown on proliferation of LN229 and U251 cells. **G**,** H** Clonogenic assay to determine effect of SelK knockdown on the proliferation of LN229 and U251 cells. **I**,** J** EdU assay to determine effect of SelK knockdown on DNA replication activity of LN229 and U251 cells. **K** Efficiency of SelK protein expression overexpression in U87 and A172 stable cell lines (Western blot). **L**,** M** ATP assay to determine effect of SelK overexpression on proliferation of U87 and A172 cells (each assay repeated three times independently). **N**,** O** Soft agar assay to determine effect of SelK overexpression on proliferation of U87 and A172 cells. **P**,** Q** Clonogenic assay to determine effect of SelK overexpression on the proliferation of U87 and A172 cells. *Significant difference at *p* < 0.05. Data are expressed as means ± SD

To investigate the potential roles of SelK in the development of GB, the expression of SelK in human normal astrocytes (NHA) and different human GB cell lines was examined using Western blots. The results indicated that the expression of SelK was higher in human GB cell lines (U87, A172, LN229, and U251) compared to NHA cells (Fig. 2A). Densitometric analysis further confirmed an obvious increase in SelK levels, specifically in LN229 and U251 cell lines. Subsequently, stable SelK knockdown cell lines were generated in LN229 and U251 cells using shRNA knockdown. The knockdown effects were confirmed by Western blot, and shSelK#1 and shSelK#2 were selected to investigate the role of SelK in GB development (Fig. 2B). The ATP (Fig. 2C, D), soft agar (Fig. 2E, F), plate cloning formation (Fig. 2G, H), and EdU (Fig. 2I, J) assays were conducted to assess the potential role of SelK in cell proliferation and tumor growth. The results showed that knockdown of SelK significantly inhibited the malignant proliferation ability of GB cells. Moreover, cell lines stably overexpressing SelK were constructed in U87 and A172 cell lines with relatively low SelK expression levels. Western blotting confirmed successful transfection efficiency (Fig. 2K). The results of ATP assay (Fig. 2L, M), soft agar assay (Fig. 2N, O), and plate colony formation assay (Fig. 2P, Q) showed that overexpression of SelK significantly enhanced the malignant proliferation of GB cells. These findings suggest that SelK may indeed play a crucial role in the development of GB cells in vitro.

### Knockdown of SelK significantly inhibits the malignant proliferative capacity of glioblastoma cells in vivo

**Fig. 3:**
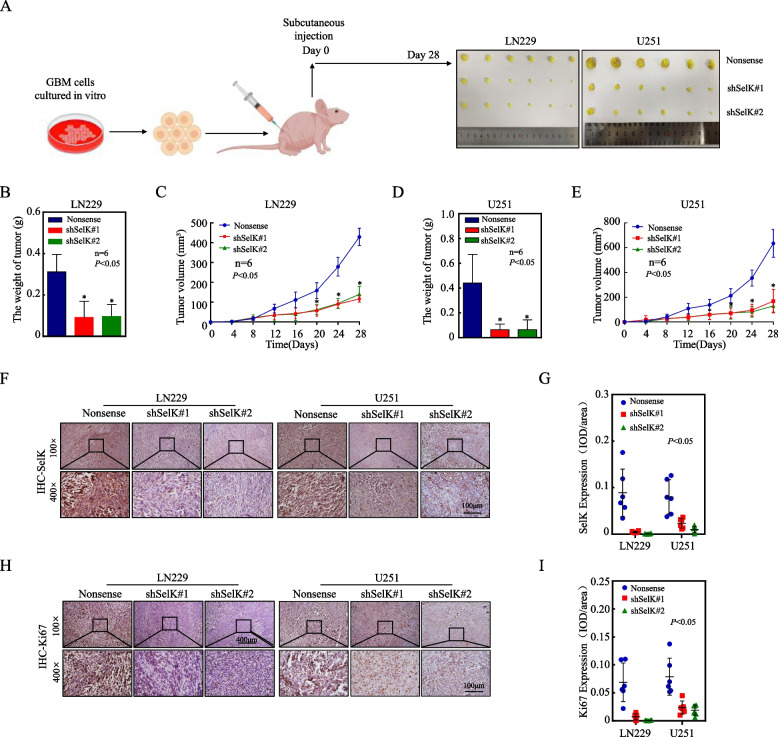
Knockdown of SelK significantly inhibited GB cell proliferative capacity in vivo.** A** GB cells LN229(Nonsense), U251(Nonsense), and knock-down SelK cells were injected into nude mice at Day 0; mice were euthanized after 28 days and tumors were isolated. **B-E** Mice after 28 days and their tumors (photographed and weighed). Tumor volume was determined every four days, and the tumor growth curves were plotted. **F**,** G** SelK protein levels in subcutaneous GB tumors heterologously-expressing SelK in nude mice (IHC). **H**,** I** Ki67 protein levels in subcutaneous GB tumors heterologously-expressing SelK in nude mice (IHC). *Significant difference at *p* < 0.05. All data are expressed as means ± SD

To evaluate the effect of SelK on the proliferative ability of GB in vivo, xenograft tumor models were established by subcutaneous injection of SelK-knockdown cells LN229, U251, or nonsense control cells into BALB/c-nude mice. After 28 days, the mice and their tumors were evaluated for growth and tumor volume (Fig. 3A). Analyses indicated that knockdown of SelK significantly inhibited the proliferative capacity of LN229 and U251 cells in vivo, which was characterized by a reduction in tumor volume and weight (Fig. 3B, E). A concurrent analysis of the proliferation marker Ki67 (IHC) in the tumors showed that the protein expression levels of SelK and Ki67 were significantly suppressed in tumor tissues that originated from SelK knockdown cells (Fig. 3F, I). These results demonstrate that knockdown of SelK may inhibit the proliferative capacity of GB cells in vivo.

###  Down-regulation of CDK4 plays a key role in knockdown of SelK-induced G 0 /G 1 phase arrest of GB cell cycle

**Fig. 4:**
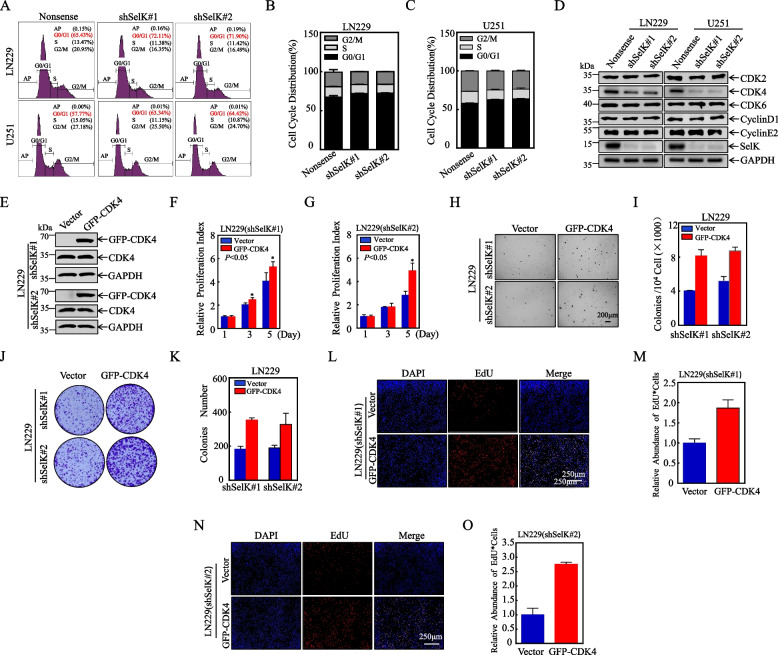
Down-regulation of CDK4 plays a key role in knockdown of SelK-induced G_0_/G_1_ phase arrest of GB cell cycle. **A-C** Flow cytometric analysis to examine cell cycle profile of LN229 and U251 cells after knockdown of SelK. **D** Levels of key proteins involved in G_0_/G_1_ phase progression (Western blot). **E** CDK4 was stably over-expressed in LN229(shSelK#1) and LN229(shSelK#2) cells and detected by Western blot. **F**, **G** Effect of CDK4 over-expression on proliferation of LN229(shSelK#1) and LN229(shSelK#2) cells using ATP assay (each assay repeated three times independently). **H**,** I** Effect of CDK4 over-expression on proliferation of LN229(shSelK#1) and LN229(shSelK#2) cells using soft agar assay. **J**,** K** LN229(shSelK#1/CDK4) and LN229 (shSelK#2/CDK4), and control cell clone formation numbers via clonogenic assay. **L-O** Effect of CDK4 over-expression on DNA replication activity in LN229(shSelK#1) and LN229(shSelK#2) (EdU assay). *Significant difference at *p* < 0.05. All data are expressed as means ± SD

Numerous signaling pathways regulate cell proliferation, including the cell cycle, which is particularly crucial. To investigate the specific molecular mechanism by which SelK might impact GB cell proliferation, flow cytometry experiments were conducted to evaluate cell cycle changes in GB cells with and without SelK knockdown. The results showed that knocking down SelK successfully arrested the GB cells in the G_0_/G_1_ phase (Fig. 4A, C).

Based on those findings, protein immunoblotting assays were employed to evaluate expressions of CDK2, CDK4, CDK6, CyclinD1, and CyclinE2, proteins implicated in G_0_/G_1_ phase cell cycle progression. Interestingly, only CDK4 protein expression was downregulated when SelK was knocked down in GB cells, implying that CDK4 may be a downstream target of SelK in the inhibition of their cell cycle (Fig. 4D). To further confirm whether CDK4 participates in cell cycle regulation by SelK, CDK4 expression was intentionally over-expressed in LN229(shSelK#1) and LN229(shSelK#2) cells (Fig. 4E). ATP (Fig. 4F, G), Soft agar (Fig. 4H, I), plate cloning (Fig. 4J, K), and EDU (Fig. 4L, O) assays performed with these cell lines showed that over-expression of CDK4 reversed the inhibitory effect of SelK knockdown on GB cell proliferative capacity. Similarly, when CDK4 was caused to be over-expressed in U251(shSelK#1) and U251(shSelK#2) cells (Figure S1A), ATP (Figures S1B-1C), soft agar (Figures S1D-1E), plate cloning (Figures S1F-1G), and EDU (Figures S1H-1 K) assays also revealed that CDK4 significantly increased the proliferative capacity of these cells. Taken together, these observations suggest that knockdown of SelK may contribute to the inhibition of GB cell proliferation, potentially through the down-regulation of CDK4 expression and the promotion of G0/G1-phase arrest.

### Knockdown of SelK promoted CDK4 ubiquitin-dependent degradation via up-regulating β-TrCP1 expression

**Fig. 5:**
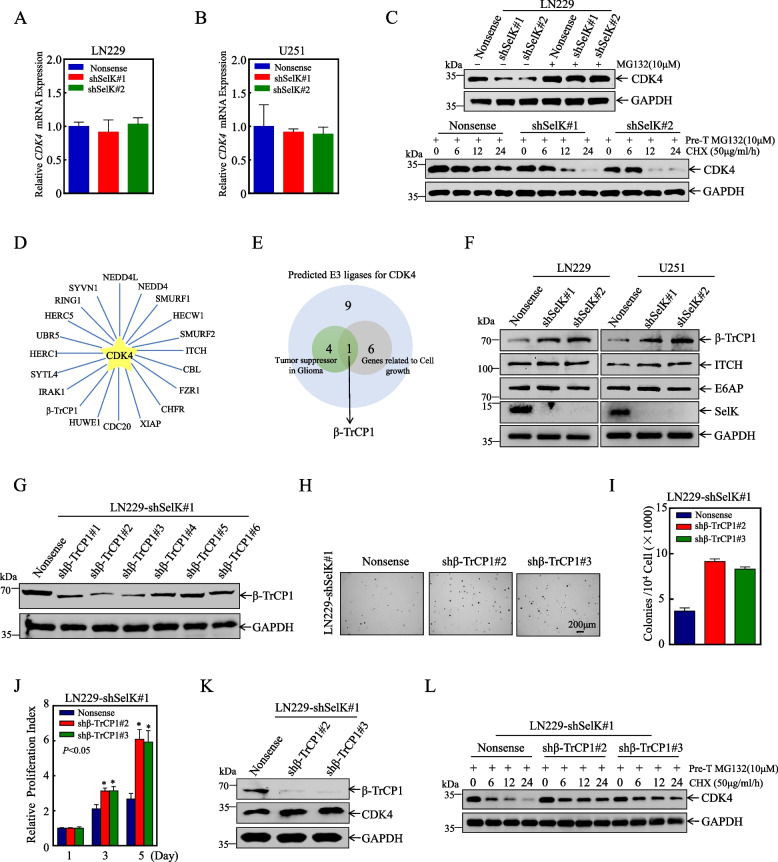
Knockdown of SelK promoted CDK4 ubiquitin-dependent degradation via up-regulating β-TrCP1 expression. **A**,** B***CDK4* mRNA level of CDK4 determined by qPCR. **C** Degradation rate of CDK4 examined by Western blot. **D** UbiBrowser database prediction of molecules that may be involved in regulating CDK4 protein degradation. **E** Venn diagram screening of E3 enzymes involved in CDK4 protein degradation regulation. **F** Protein levels of β-TrCP1 and E3-ligases known to target CDK4 (Western blot). **G** Knockdown efficiency of β-TrCP1 (Western blot). **H**, **I** Effect of β-TrCP1 knockdown on cell proliferation of LN229(shSelK#1) (soft agar assay).** J** Effect of β-TrCP1 knockdown on cell proliferation of LN229(shSelK#1) (ATP assay).** K** Analysis of CDK4 levels in LN229(shSelK#1/shβ-TrCP1 #2 and #3), and LN229 (shSelK#1/Nonsense) cells (Western blot). **L** CDK4 protein degradation following β-TrCP1 knockdown (Western blot). *Significant difference at *p* < 0.05. All data are expressed as means ± SD

To explore the potential role of SelK on the regulation of CDK4 expression, qPCR experiments were conducted to analyze the effects of SelK knockdown on *CDK4* mRNA levels in LN229 and U251 cells. The results showed that SelK had no effect on the regulation of *CDK4* mRNA (Figs. 5A-B). This phenomenon indicated that SelK may regulate CDK4 expression in a post-transcriptional manner. The ubiquitination-proteasome degradation pathway is the primary post-transcriptional regulatory mechanism for proteins in eukaryotic cells. To investigate whether this pathway is involved in SelK's regulation of CDK4 protein levels, cells were pre-treated with the proteasome inhibitor MG132. The results showed that following MG132 treatment, there was a noticeable accumulation of CDK4 in SelK knockdown cells. After CDK4 accumulated to similar levels in SelK Nonsense control cells and SelK knockdown cells, MG-132 was removed, and the protein synthesis inhibitor cycloheximide (CHX) was added for varying durations to assess the CDK4 degradation rate. The results indicated that SelK knockdown notably accelerated the ubiquitination and degradation of CDK4 (Fig. 5C). To examine the potential mechanism by which SelK regulates CDK4, ITCH (an E3 ubiquitin ligase of CDK4 that destabilizes CDK4 and inhibits CRC cell survival [29]) and E6AP (which ubiquitin CDK4 in HEK293 cells as determined by orthogonal ubiquitin transfer (OUT) method [30]) were also evaluated in the cell lines. In addition, a UbiBrowser database search was conducted to analyze for other possible E3 ligases involved in CDK4 ubiquitin-dependent degradation (Fig. 5D, E). The results showed that knockdown of SelK in LN229 and U251 cells increased only β-TrCP1 expression, while the levels of other E3 ligases, ITCH and E6AP, remained relatively unchanged (Fig. 5F). Taken together, these data suggested SelK regulated CDK4 expression and cell proliferation, in part, via β-TrCP1 mediated CDK4 protein degradation.

To further clarify whether CDK4 is regulated by the potential E3 enzyme β-TrCP1 and whether β-TrCP1 is involved in the malignant proliferation of GB. LN229(shSelK#1) cells with stable knockdown of β-TrCP1 were constructed (Fig. 5G), and the effects of β-TrCP1 knockdown on cell proliferation were examined in soft agar assays (Fig. 5H-I) and ATP (Fig. 5J). The results showed that β-TrCP1 knockdown rescued the inhibitory effects of SelK knockdown on LN229 cell growth under both monolayer and anchorage-independent conditions. The effects of β-TrCP1 knockdown on CDK4 protein expression were also evaluated (Western blot) and protein degradation assays. The results indicated that knocking down β-TrCP1 resulted in a slight but observable increase in CDK4 levels and a decrease in the degradation rate of CDK4 in LN229(shSelK#1) cells (Fig. 5K, L). These findings suggest that β-TrCP1 plays a pivotal role in SelK-mediated GB cell proliferation and CDK4 protein expression.

### β-TrCP1 directly interacts with and ubiquitinates CDK4

**Fig. 6:**
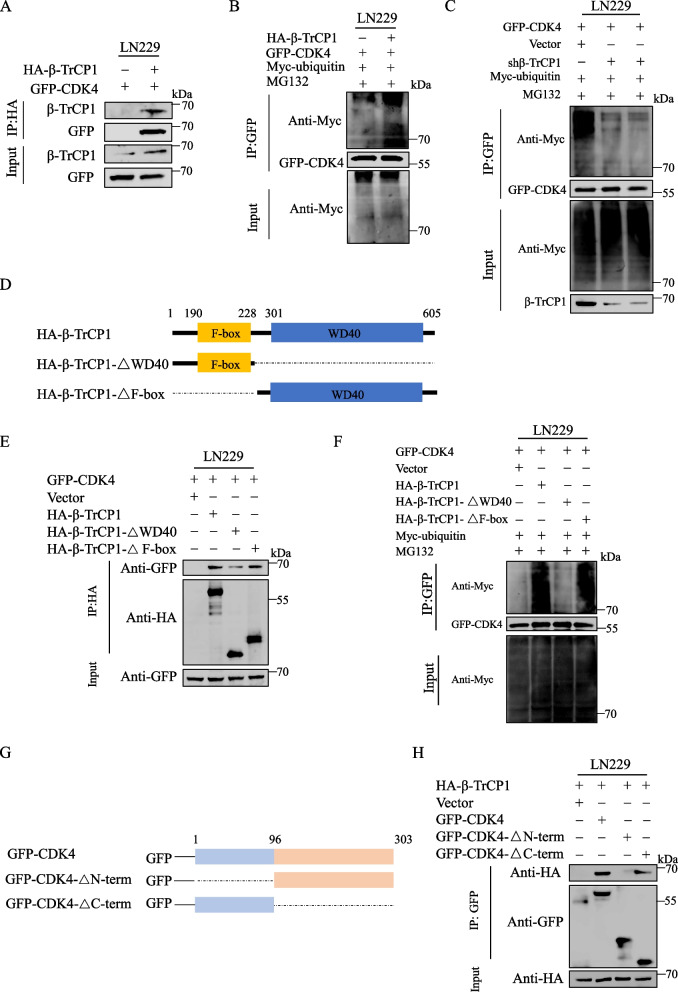
β-TrCP1 directly interacts with and ubiquitinates CDK4. **A** Co-IP assay to verify relationship between β-TrCP1 and CDK4 in LN229 cells. **B** Ubiquitination process from β-TrCP1 to CDK4 was detected in LN229 cells. **C** Effect of β-TrCP1 knockdown on CDK4 ubiquitination in LN229 cells (ubiquitination assay). **D** Schematic diagram of HA-β-TrCP1 structural domain deletion construct. **E** Co-IP assay to detect interaction between CDK4 and β-TrCP1 structural domains in LN229 cells. **F** Ubiquitination assay to detect ubiquitination between CDK4 and β-TrCP1 structural domains in LN229 cells. **G** Schematic diagram of GFP-CDK4 structural domain deletion construct. **H** Co-IP assay to detect interaction between β-TrCP1 and CDK4 structural domains in LN229 cells

To identify the E3 Ub ligase β-TrCP1 that interacts with CDK4, HA-β-TrCP1 and GFP-CDK4 plasmids were co-transfected into LN229 cells that, in turn, under-went co-immunoprecipitation (Co-IP) analysis. The results indicated that tagged β-TrCP1 directly interacted with CDK4 (Fig. 6A) and promoted its ubiquitination status (Fig. 6B) in LN229 cells. Further, knockdown of β-TrCP1 by shRNA decreased the ubiquitination of GFP-CDK4 (Fig. 6C). Hence, the data clearly indicated that β-TrCP1 not only directly interacted with CDK4 protein but also mediated its ubiquitination.

To more fully flesh out the mechanisms of action involved, the CDK4-interacting domains in β-TrCP1 were mapped, and truncated mutants of β-TrCP1 were subsequently generated. As shown in Fig. 6D, the *N*-terminal domain contained an F-box motif, and the *C*-terminal domain contained seven WD repeats (WD40). Co-expression of full length β-TrCP1 and two deletion constructs with CDK4 in LN229 cells showed that the WD40 region is mainly bound to CDK4. (Fig. 6E). Further, deletion of the WD40 domain from aa 301–605 from β-TrCP1 (β-TrCP1-ΔWD40) apparently reduced the ubiquitination of GFP-CDK4 compared with that of GFP-CDK4 upon its co-expression with full-length β-TrCP1 or other deletion mutants examined (. 6F). In addition, as shown in Fig. 6G, a GFP-CDK4 structural domain deletion was also successfully constructed that lacked an *N*-terminal (amino acids 1–96) and *C*-terminal (amino acids 96–303) region. Immunoprecipitation experiments revealed that only the *N*-terminal region of CDK4 would bind to β-TrCP1 with HA tags (Fig. 6H). Collectively, these findings indicated that the interaction between CDK4 and β-TrCP1 is primarily mediated through a β-TrCP1 WD40 structural domain and a CDK4 *N*-terminal region, ultimately supporting the ubiquitination modification of CDK4.

### SelK up-regulates SKP2 and promotes SKP2-mediated β-TrCP1 ubiquitination

**Fig. 7:**
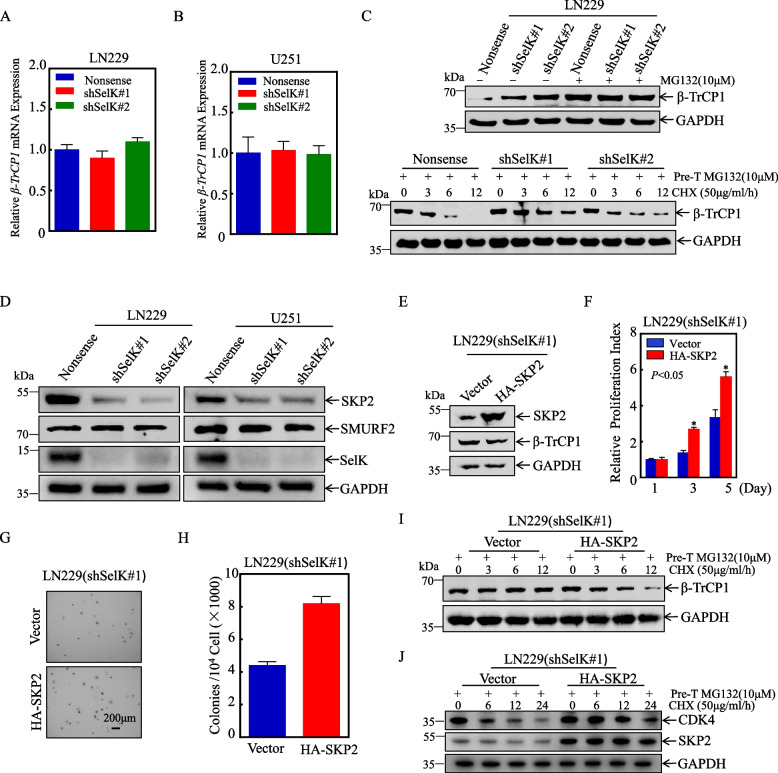
SelK up-regulates SKP2 and promotes SKP2-mediated β-TrCP1 ubiquitination. **A**,** B***β-TrCP1* mRNA level determined by qPCR. **C** Degradation rate of β-TrCP1 protein examined by Western blot. **D** Protein levels of known E3-ligases that target β-TrCP1 (Western blot). **E** Verification of efficiency of stable SKP2 over-expression and β-TrCP1 protein expression after SKP2 over-expression (Western blot). **F** ATP assay to examine effect of SKP2 over-expression on malignant proliferation of LN229(shSelK#1) cells (each assay repeated three times independently). **G**,** H** Effect of SKP2 over-expression on malignant proliferation of LN229(shSelK#1) cells (soft agar assay). **I** β-TrCP1 protein degradation after SKP2 over-expression (Western blot). **J** CDK4 protein degradation after SKP2 overexpression (Western blot). *Significant difference at *p* < 0.05. All data are expressed as means ± SD

The above results indicated that β-TrCP1 plays a crucial role in the growth of GB cells, serving as a key regulator of CDK4 under the influence of SelK. Additionally, quantitative PCR analysis revealed that SelK primarily regulated β-TrCP1 at the protein level, as there was no obvious change in the transcript levels of β-TrCP1 in GB cells with SelK knockdown (Fig. 7A, B).

To further probe the impact of SelK on GB cell growth progression, protein degradation assays were performed to assess the effects of SelK on β-TrCP1 stability. The assay results demonstrated that β-TrCP1 was more stable in cells with SelK knockdown than in control cells, indicating that SelK inhibited the degradation rate of β-TrCP1 (Fig. 7C). Previous studies have implicated SKP2 and SMURF2 in the ubiquitination modification of β-TrCP1 [31, 32]. Therefore, expressions of SKP2 and SMURF2 in GB cells with SelK knockdown were assessed (Western blot). The data showed that while there was no clear alteration in SMURF2 protein levels, SKP2 levels were consistently down-regulated in these cells (Fig. 7D). Thus, this data suggests that SelK may promote CDK4 protein expression by increasing SKP2 protein levels and reducing β-TrCP1 expression.

To further investigate the relationship between SKP2 and β-TrCP1 and to determine whether SelK-mediated regulation of SKP2 influences malignant GB proliferation, SKP2 was made to be over-expressed in LN229 (shSelK#1) cells and in a stable cell line. Interestingly, β-TrCP1 protein levels were reduced in SelK-knockdown cells with SKP2 over-expression (Fig. 7E). Further analysis revealed that SKP2 over-expression led to increased proliferation of LN229 (shSelK#1) cells compared to control cells, as shown in both ATP and soft agar assays (Fig. 7F, H). Protein degradation assays demonstrated that β-TrCP1 protein turnover was higher in cells with SelK knockdown and SKP2 over-expression compared to control cells (Fig. 7I). Additionally, protein degradation assays demonstrated that CDK4 was more stable in cells with SelK knockdown and SKP2 over-expression compared to control cells (Fig. 7J). Together, these results collectively imply that SelK enhances β-TrCP1 ubiquitination by augmenting protein levels of SKP2, leading to decreased β-TrCP1 protein expression and increased CDK4 protein expression, ultimately promoting GB cell proliferation.

### Knockdown of SelK down-regulates SKP2 by activating ER stress

**Fig. 8:**
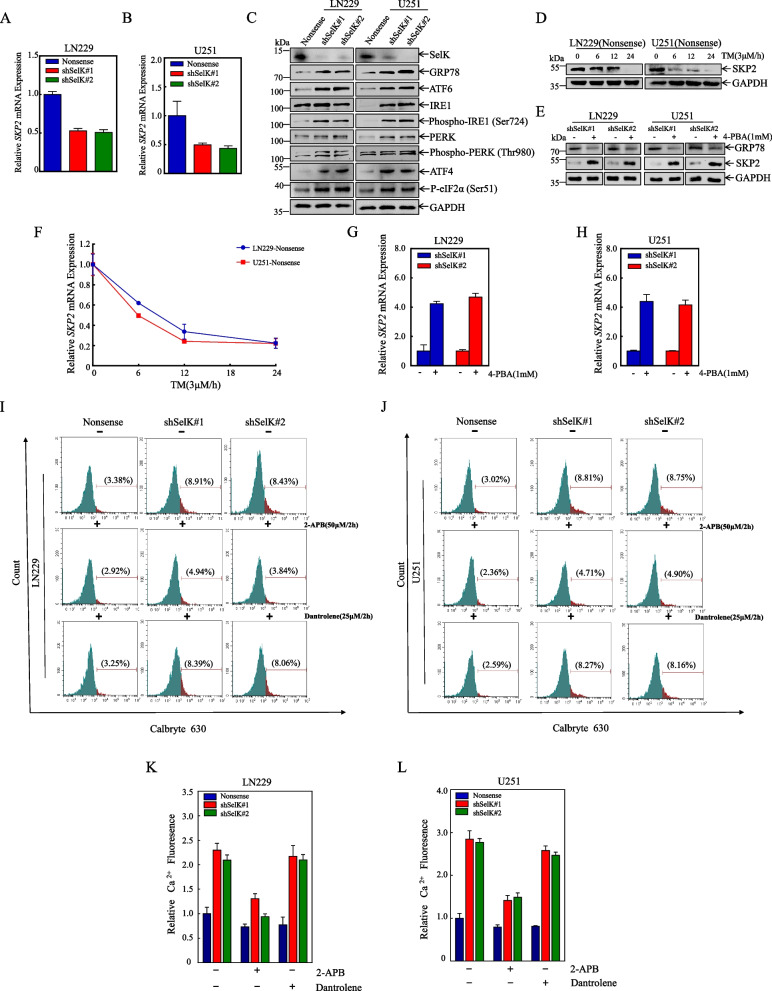
Knockdown of SelK activates ER stress by releasing calcium through IP3R channels, thereby down-regulating SKP2. **A**,** B***SKP2* mRNA levels determined by qPCR. **C** Detection of major markers of endoplasmic reticulum stress (Western blot).** D** SKP2 protein expression was detected in LN229 (Nonsense) and U251 (Nonsense) cells after treatment with 3 μM TM for 0, 6, 12 and 24 h. **E** The protein expression of SKP2 and GRP78 were detected after treatment of LN229 (shSelK#1), LN229 (shSelK#2), U251 (shSelK#1) and U251 (shSelK#2) cells with 1 mM 4-PBA for 12 h.** F** LN229 (Nonsense) and U251 (Nonsense) cells were treated with 3 μM TM for 0, 6, 12, and 24 h, and then *SKP2* mRNA levels were assessed (qPCR). **G**,** H***SKP2* mRNA levels measured by qPCR after 12 h of 1 mM 4-PBA exposure of LN229 (shSelK#1), LN229 (shSelK#2), U251 (shSelK#1), and U251 (shSelK#2) cells. **I-L** Intracellular calcium levels in LN229 and U251 cells with knockdown SelK and in counterpart control cells evaluated using Calbryte 630 staining, after pre-treatment with 2-APB (50 μM) and dantrolene (25 μM) for 2 h, and flow cytometry

To further clarify how SelK promotes SKP2 protein expression at a molecular level, real-time PCR assays were conducted to examine *SKP2* mRNA levels in cells with SelK knockdown and in control cells. The results showed a significant decrease in *SKP2* mRNA levels after SelK knockdown in LN229 and U251 cells, which was consistent with a decrease in SKP2 protein expression (Fig. 8A, B). As previously reported, SKP2 is down-regulated during ER stress and is not dependent on proteasome-mediated protein degradation but, rather, through transcriptional regulation of SKP2 [33]. To examine this, levels of ER stress-related proteins GRP78, ATF6, IRE1, PERK, ATF4, and P-eIF2α [34] were examined in cells with SelK knockdown and in control cells (Western blot). Because IRE1 and PERK are functionally active in a phosphorylated form, the corresponding phosphorylated forms were also evaluated. The results showed an increase in each of these ER stress-related proteins in SelK knockdown of LN229 and U251 cells compared to the control group (Fig. 8C). This suggested that a decrease in SelK leads to an increase in ER stress-related proteins and thus increased ER stress.

To confirm the relationship between SKP2 and ER stress, the ER stress inducer tunicamycin (TM) and the ER stress inhibitor 4-phenylbutyrate (4-PBA) were employed. The results showed that there was a decrease in SKP2 protein expression with increasing TM duration (Fig. 8D). SKP2 protein expression was also affected in SelK knockdown of LN229 and U251 cells that had been treated with 4-PBA for 12 h. The results showed that the expression of GRP78 protein was down-regulated, and that of SKP2 protein was up-regulated compared to that of non-treated cells after inhibiting ER stress by knocking down SelK (Fig. 8E).

Lastly, measures of *SKP2* mRNA levels using real-time PCR after treating LN229 (Nonsense) and U251 (Nonsense) cells with TM found that the treatment reduced *SKP2* mRNA levels in a time-dependent manner (Fig. 8F). Further, inhibiting ER stress using 4-PBA significantly increased *SKP2* mRNA levels, as measured using real-time PCR, in SelK knockdown of LN229 and U251 cells (Fig. 8G, H). In conclusion, SelK knockdown induced ER stress, leading to decreased SKP2 protein via transcriptional regulation.

### Knockdown of SelK activates ER stress by releasing calcium through IP3R channels

To assess whether SelK induces ER stress through regulation of Ca^2+^ channels, dantrolene and 2-aminoethoxydiphenyl borate (2-APB), RyR, and IP3R channel inhibitors were used to study cell effects [35, 36]. In cells with knockdown SelK, free Ca^2+^ ion levels were increased compared to those in the control group. Treatment with 2-APB resulted in a decrease in the intracellular Ca^2+^ levels in GB cells, which was caused by the inhibition of IP3R channels, leading to a decreased release of Ca^2+^ ions from the ER to the cytoplasm. However, dantrolene treatments did not perform equivalently (Fig. 8I, L). To further investigate the effects of SelK depletion mediated by the IP3R channels, we assessed whether 2-APB could rescue the impact of SelK knockdown on CDK4 degradation and GB cell proliferation. Results from protein degradation experiments indicated that 2-APB treatment slowed down the rate of CDK4 protein degradation (Figure S2A), and ATP and plate cloning formation assays demonstrated that 2-APB treatment reversed the growth inhibitory effect of SelK (Figure S2B-2D). The experimental results suggest that SelK may induce ER stress by affecting IP3R channels, which leads to a change in levels of intracellular Ca^2+^ ions.

### Correlation between SelK, CDK4, and β-TrCP1 protein levels in clinical samples

**Fig. 9:**
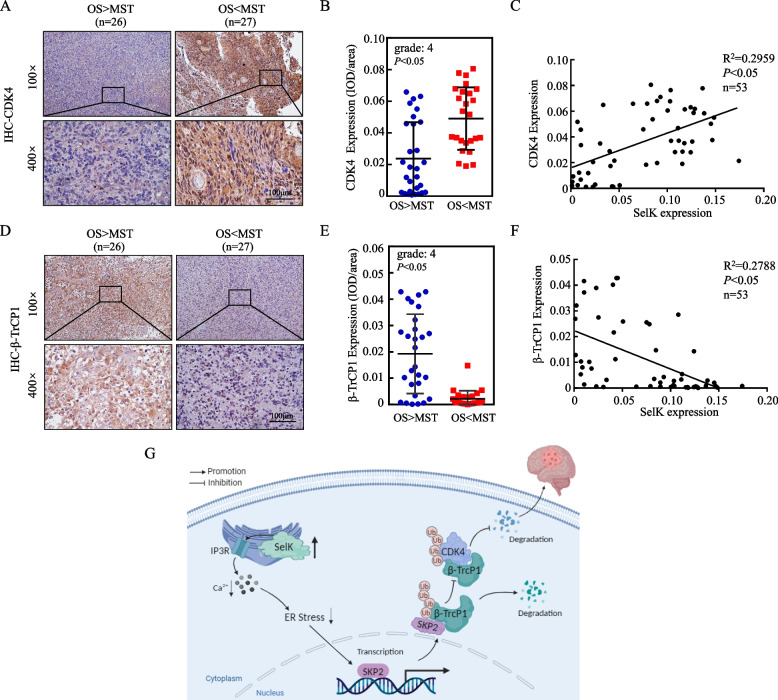
Correlation between SelK, CDK4, and β-TrCP1 protein levels in clinical samples. **A**,** B** CDK4 expression (IHC). **C** Correlation analysis of SelK and CDK4 expression. **D**,** E** β-TrCP1 expression (IHC). **F** Correlation analysis of SelK and β-TrCP1 expression. **G** Graphical abstract. *Significant difference at *p* < 0.05. All data are expressed as means ± SD

To further explore the expression of SelK and its downstream genes with respect to expression correlation, we initially analyzed the expression levels of CDK4, β-TrCP1, and GRP78 in nude mice tumor tissue samples using IHC. The results showed that GRP78 and β-TrCP1 were notably increased in SelK knockdown tumors, and CDK4 was significantly decreased (Figure S3). Subsequently, we assessed the expression levels of CDK4 and β-TrCP1 in GB tissue samples (IHC). IHC quantitative analysis showed CDK4 was significantly up-regulated in tissue samples from GB patients with short survival periods (Fig. 9A, B), and β-TrCP1 was significantly down-regulated in tissues from GB patients with short survivals, as compared to relative expression levels in samples from GB patients with long survival periods (Fig. 9D, E). Overall, while the correlation observed in Fig. 9F is moderate, SelK expression was positively correlated with the expression of CDK4 and negatively correlated with that of β-TrCP1(Fig. 9C, F). The weak correlation between SelK and β-TrCP1 may be attributed to the limited number of clinical samples available for analysis. The above results suggest that regulation of downstream genes by SelK has some clinical generality.

Based on the totality of all the studies performed here, a potential mechanism by which knockdown of SelK inhibits malignant proliferation of GB cells could now be formulated (Fig. 9G).

## Discussion

Advances in genomics technology have improved the understanding of the major molecular changes that contribute to the development of GB. It is well known that patients with IDH-wildtype GB have an overall worse prognosis; however, prognoses still vary considerably among these patients, and the mechanisms involved are still unclear. Therefore, exploring the molecular mechanisms involved in long-survival GB will help define novel prognostic biomarkers and potentially new therapeutic targets. In this study, by quantitative proteomic analysis of whole protein TMT labeling, we found that in GB, patients with shorter survival time (≤ 1 year) had higher expression of SelK compared to tumors of patients with longer survivals (≥ 3 years). This suggests that SelK expression is strongly associated with the GB prognosis. Through cellular experiments and nude mouse subcutaneous tumor models, our study illustrated that SelK significantly enhanced the malignant proliferation of GB cells. However, our experiments have certain limitations. For instance, the use of nude mouse subcutaneous tumor models may not fully capture the complexity of the natural tumor microenvironment. Alternatively, injecting cells directly into the brain could offer more relevant interactions with the brain matrix. Moreover, utilizing cell lines with inducible shRNA and activating the shRNA through doxycycline administration in animals can help mitigate the impact of gene knockout on GB cell engraftment, thereby enabling a more accurate assessment of gene effects on tumor cells.

Our study found that SelK promotes GB cell cycle and proliferation through the ER stress/SKP2/β-TrCP1/CDK4 pathway. In the cell cycle assay, we found that SelK knockdown has an impact on the cell cycle. Although the observed change is small, we believe that even minor effects on the cell cycle can accumulate and ultimately have a significant influence on tumor growth. CDK4, in particular, serves as an important competitive target for drug inhibitors. Previous studies have shown that CDK4 is upregulated in a variety of tumors and that inhibition of CDK4 expression improves clinical management of melanoma, breast cancer, GB, and liposarcoma [37–40]. During cell division, activated CyclinD-CDK4/6 promotes transcription of cell cycle-related genes by phosphorylating the substrate RB, which prompts the separation of RB from E2F1/2/3 and activates the transcriptional activity of E2F1/2/3 to ensure the G1-S phase transition [41]. We found that SelK specifically upregulates CDK4 protein expression and promotes the transition from G0/G1 to S phase in GB cells. Inhibition of SelK expression can indirectly inhibit CDK4 expression, and these events suggest to us that SelK may be a potential new target for the treatment of GB.

β-TrCP1 is an E3-ubiquitin ligase that affects various tumorigenesis and development processes by targeting ubiquitinated substrates [42]. Previous studies have found significantly lower levels of β-TrCP1 protein expression in gliomas compared to non-tumor brain tissue, and even further, the higher the grade of glioma, the lower the level of β-TrCP1 expression [43]. In this study, we explored the mechanism leading to the down-regulation of CDK4 expression in GB and revealed that SelK knockdown up-regulated β-TrCP1 expression. In addition, this study demonstrated for the first time that β-TrCP1 can directly bind and interact with CDK4, leading to the degradation of CDK4 protein. β-TrCP1 plays a tumor suppressor role in GB and may serve as a potential new target for GB therapy.

In previous studies, SelK was found to regulate ER stress in hepatocellular carcinoma cells [25], foam cell formation, and atherogenesis by regulating CD36 palmitoylation on the surface of macrophages [44]. Based on that information, the present study examined the protein expression of three major pathways in the onset of ER stress in GB cell lines and found that knockdown of SelK induced ER stress in both lines. Further, the study here showed that SKP2 protein expression was down-regulated in cells without knockdown of SelK after treatment with TM and up-regulated in cells with knockdown of SelK after treatment with 4-PBA. These results suggest that ER stress regulates SKP2 protein expression by affecting its mRNA levels. The data here indicated there was a decreasing trend in *SKP2* mRNA levels after the induction of ER stress and a consistently up-regulated trend after the inhibition of ER stress. However, the specific mechanism of action is not yet clear. Undoubtedly, further studies are needed.

Lastly, SelK is known to promote calcium flow and reduce calcium ion levels in cells in vivo after immune cell activation [45]. At the same time, knockdown of SelK in neuronal cells induces an increase in calcium ion levels, which leads to ER stress and apoptosis [24]. Knockdown of SelK in this study increased the intracellular level of free calcium ions in GB, which caused the onset of ER stress and down-regulated SKP2 protein expression. Furthermore, the present study confirms that SelK activates ER stress in these cancer cells by affecting IP3R channels and regulating Ca^2+^ ion homeostasis in the ER.

In conclusion, this study demonstrated the important role of a SelK/ER stress/SKP2/β-TrCP1/CDK4 axis in GB proliferation. From the findings here, it seems clear that SelK is an important oncogenic molecule in the development and/or maintenance of GB cells. Thus, the development of small molecule inhibitors or agonists that could target SelK and its downstream effectors will likely help guide treatment strategies for GB patients.

## Materials and methods

### Proteomic analysis

Biospecimens were collected from newly diagnosed patients who underwent surgical resection (The First Affiliated Hospital of Wenzhou Medical University, Zhejiang, China). All sample collection procedures complied with routine clinical practice. Protein sample preparation and liquid chromatography-tandem mass spectrometry (LC–MS/MS) using the Tandem Mass Tag (TMT) were performed at PTM Biolab Co., Ltd. (Hangzhou, Zhejiang, China). Briefly, cells were harvested to fetch whole proteins, which were further proceeded to digestion. After the trypsin digestion procedure, peptides were labeled by TMT/iTRAQ according to the manufacturer’s protocol for TMT kit/iTRAQ kit. The tryptic peptides were fractionated into fractions by high pH reverse-phase HPLC using Thermo Betasil C18 column (5 μm particles, 10 mm ID, 250 mm length). These peptides were subjected to NSI source followed by tandem mass spectrometry (MS/MS) in Q ExactiveTM Plus (Thermo) coupled online to the UPLC. MS data were processed using Proteome Discoverer 1.3.

### Plasmids, reagents, and antibodies

SelK knockdown plasmid and its control plasmid, as well as β-TrCP1 knockdown plasmid and its control plasmid, were purchased from Open Biosystems Company (Huntsville, USA). The PEGFP-CDK4 over-expression plasmids were constructed on-site as described in previous studies [46, 47]. HA-SKP2 over-expression plasmid, HA-β-TrCP1 over-expression plasmid, HA-β-TrCP1-C-term plasmid, HA-β-TrCP1-N-term plasmid, GFP-CDK4-N-term plasmid and GFP-CDK4-C-term plasmid were constructed on-site. MG132 and cycloheximide (CHX) were bought from Calbiochem (San Diego, CA, USA). Antibodies specific against SelK (PA5-52,529) were purchased from Invitrogen (Grand Island, NY, USA). Antibodies specific against CDK2 (Sc-6248), CDK4 (Sc-260), CDK6 (Sc-177), cyclinD1 (Sc-20044), cyclinE2 (Sc-481), GFP (Sc-9996), E6AP (Sc-166689), GRP78 (Sc-13539), and SMURF2 (Sc-518164) were purchased from Santa Cruz Biotechnology (Santa Cruz, CA, USA). Antibodies specific against β-TrCP1 (11984S), SKP2 (2652S), P-PERK (3179S), HA (3724S), Myc (2276S), ATF4 (11815S), and P-eIF2α (Ser51,3597S) were obtained from Cell Signaling Technology (Boston, MA, USA). Antibodies specific against IRE1 (DF7709) and P-IRE1 (Ser 724, AF7150) were obtained from Affinity. Antibody specific against GAPDH (10,494–1-AP) was obtained from Proteintech (Chicago, IL, USA).

### Western blot analysis

Aliquots of cells (10^6^) were lysed on ice for 30 s-1 min using cell lysis buffer Boiling Buffer (containing 10% SDS, 100 mM Na_3_VO_4_, 1 M Tris–HCl [pH7.4]), followed by sonication. After centrifugation for 10 min, the concentration of protein in the supernatant was measured using a NanoDrop One system (Thermo Fisher Scientific, USA). Each protein sample was homogenized, and equal amounts of protein were loaded into each well on 10% or 12% SDS-PAGE gels and the proteins were then resolved. Gel contents were then electrotransferred to a PVDF membrane. Each membrane was then incubated in a solution of TBS (Tris-buffered saline, pH 7.4) containing 5% skim milk powder for 60 min at room temperature. Dedicated membranes were generated for each protein of interest to negate any need for membrane stripping for re-analysis of another protein.

Each membrane was then placed in a solution of TBST (TBS-0.1% Tween-20) bearing a specific primary antibody (at manufacturer-recommended dilution) and incubated overnight at 4 °C with gentle shaking. The following day, each membrane was rinsed three times with TBST, then placed in a solution of 5% skim milk containing the specified secondary antibody (at manufacturer-recommended dilution) and incubated at 4 °C for 4 h. After a final rinsing with TBST, each membrane was treated with developing solution to visualize all bound antibody. In all cases, glyceral-dehyde-3phosphate dehydrogenase (GAPDH) expression was assessed to monitor for gel loading. Ultimately, all membranes were scanned using a Typhoon FLA 7000 phosphor imager system (GE Healthcare). All protein expression values were then normalized to GAPDH levels.

### Clinical specimens, cell culture, and transfections

A total of 88 clinical GB samples were provided by the First Affiliated Hospital of Wenzhou Medical University (Zhejiang, China). Collection of all samples had been approved by the Clinical Research Ethics Committee of The First Affiliated Hospital of Wenzhou Medical University (permission: 2023-R262). Astrocytes NHA were purchased from ScienCell (San Diego); human GB cells U87, A172, LN229, and U251 cells were bought from ATCC (Manassas, VA). All lines were confirmed by STR typing without errors. U87 and U251 cells were cultured in minimum essential medium (MEM; Gibco, #11,095–080). NHA, A172, and LN229 cells were cultured in Dulbecco’s modified Eagle’s medium (DMEM; Gibco, #11,995–065). All media contained 10% fetal bovine serum (FBS, Gibco, #10,437–028). All cells were cultured in 37 °C incubators with 5% CO_2_. All stable cell lines were generated by lentiviral infection and infection for ectopic expression. Lentiviral infection was performed when the HEK293 cell density reached 70–80%, using transfection reagent 1.2 μg psPAX2 (Addgene, #12,260), 1.2 μg pMD2.G (Addgene, #12,259), and 2.0 μg target plasmid transfected into HEK293 cells. After 48 h, the medium containing viral particles was collected and centrifuged, and the supernatant was filtered and used to infect cells. Ectopic expression infection was a PolyJet™ DNA in vitro Transfection reagent (SignaGen Laboratory, SL100688) for transfecting plasmid into LN229 and U251 cells, and G418 (4000-5000ug/mL for LN229, 1000–2000 5000ug/mL for U251, Santa Cruz, sc-29065) was used to select cells stably expressing corresponding resistance constructs.

### Soft agar colony formation assay (soft agar)

A lower gel (containing 0.5% soft agar in 10% FBS-basal medium Eagle [BME]) was prepared first in the bottom of 6-well culture plates; after it solidified, an upper gel (10% FBS-BME containing 0.33% agar) containing the quantitative cells (i.e., 10^4^ cells) was overlayed. The plates were then incubated at 37 °C in 5% CO_2_ for 2–3 wk. Soft agar experiments were performed in three wells of a 6-well plate, with five independent areas photographed from each well using a microscope equipped with a camera; all clones containing > 32 cells were counted and analyzed. Representative images were selected based on the clone count results.

### Quantitative real-time PCR (qRT-PCR)

Total RNA was extracted from 5 × 10^5^ cells using TRIzol reagent (Invitrogen, #15596018). After quantification of the total RNA concentration using a BioDrop μLite spectrophotometer (BioDrop, Cambridge, UK), cDNA was generated by PCR using reverse transcription reagents (Takara, #RR037A). All procedures were performed according to manufacturer instructions. Then each sample was subjected to three wells in a 384-well plate and analyzed using the Q6 real-time PCR system (Thermo Fisher Scientific, Waltham, MA, USA). The qPCR procedures were described in detail in a previous study [48], and the primers used are noted in Supplementary Table 3.

### Nude mouse xenograft model

This study was approved by the Laboratory Animal Ethics Committee of Wenzhou Medical University. BALB/c nude mice (female, 3–4 wk-of-age) were obtained from GemPharmatech (Nanjing, Jiangsu, China) and housed in an SPF-level experimental area maintained at 20–26 °C with a 40–70% relative humidity at the Animal Center of Wenzhou Medical University. All mice had ad libitum access to standard rodent chow and filtered tap water. Mice were housed for ~ 1 wk and then randomly allocated into three groups for treatments with Nonsense, shSelK#1 and shSelK#2, respectively (*n* = 6/group).

LN229 and U251 knockdown and control cells were stably transfected and identified for animal experiments. Into the right flank of each mouse, a total of 3 × 10^6^ LN229 (Nonsense), LN229 (shSelK#1), LN229 (shSelK#2), U251 (Nonsense), U251 (shSelK#1) and U251 (shSelK#2) cells were subcutaneously injected into nude mice. After ~ 3 wk, when the tumor had grown to an appropriate size, all the mice were euthanized. At autopsy, the tumors were removed, measured in volume and weighed, and then photographed.

### ATP assay

Cells were seeded into 96-well plates at a density of 1.5 × 10^3^ cells per well, with each cell type represented in five wells. The cells were cultured in medium containing 0.1% FBS at 37 °C for 12 h. Subsequently, the medium was removed and replaced with medium containing 10% FBS for a varying number of days. After the defined incubation period, the medium in each well was removed, and equal amounts of ATP reagent (G7572, Promega) and PBS (phosphate-buffered saline, pH 7.4) were added to each well. The reagents were mixed, and after 10 min at room temperature, the mixture was measured for total fluorescence in a Centro LB 960 luminometer (Berthold Technologies, Berthold, Germany), and each assay was repeated 3 times independently.

### Ubiquitination assay

Instantaneous transfection of ubiquitin and substrate granules into cells in a 3:1 ratio. After 24 h, ubiquitin was co-expressed with the substrate in cells; at that point, 10 μM MG132 was added and the cells were incubated a further 8 h. Co-immunoprecipitation was then performed as described above.

### Intracellular Ca^2+^ concentration measurement

Cells were harvested from their culture dishes and prepared as suspensions in 1.5 ml tubes. Each tube then received 500 μL HBSS and 0.5 µl (5 mM) Calbryte™ 630 AM (AAT Bioquest, Sunnyvale, CA, USA) and was incubated for 1 h at room temperature, according to manufacturer instructions. The cells were then washed twice with HBSS to remove excess dye and were immediately analyzed using the CytoFLEX flow system three times to measure fluorescence intensity.

### EdU assay

For these studies, cells were evaluated using an EdU Assay Kit (Ribobio, Guangzhou, China; #c10310-2). In brief, LN229 and U251 cells (10^4^ cells/well) were plated into 96-well plates, with each cell type seeded in three wells. After 24 h of incubation, the medium in each well was removed and replaced with 100 μL EdU medium (50 μM) and incubated for a further 2 h. The cells were then fixed with 50 μL 4% paraformaldehyde/well, and then treated with 50 μL of a 2 mg glycine/ml solution. The cells were then treated with 100 μL kit-provided penetrant (PBS containing 0.5% TritonX-100), and incubated for 10 min at 37 °C. Thereafter, 100 μL kit-provided Apollo® staining reaction solution was added to each well. The cells were then examined using a fluorescence microscope and images captured for quantification of EdU staining (following manufacturer protocols and scoring recommendations).

### Protein degradation experiment

Aliquots of cells (80–90%) were inoculated into 6-well plates and incubated in medium containing 0.1% FBS for 12 h, followed by 8 h of incubation first in a medium containing 10 μM MG132/10% FBS. The cells were then treated with a medium containing 50 μg cycloheximide (CHX)/ml and 10% FBS for 0, 6, 12, or 24 h or for 0, 3, 6, 12 h. Finally, the cells were collected and their proteins were isolated for Western blot analyses.

### Flow cytometry

Aliquots of the test cells (10^6^ cells in 2 ml medium) were inoculated into 6-well plates and incubated—sequentially—with medium containing 0.1% FBS for 12 h and then with medium bearing 10% FBS for 12 h. After that, the cells were removed from the plates using trypsin and transferred into Eppendorf tubes wherein they were then fixed overnight at 4 °C with 70% pre-cooled alcohol. Then, after pelleting the cells, to each tube, 40 μL of RNase A and 360 μL of PI were then added and the cells were incubated at room temperature for 30 min. The cells were then examined for cell cycle status in a CytoFLEX system (Beckman Coulter) for three times. The steps were described in detail in our previous study [49].

### Immunohistochemistry

Immunohistochemical (IHC) staining was used to analyze levels of specific proteins in the 88 clinical GB samples and in mouse samples. A commercial kit (Boster Bio, #SA1022) that contained 3% H_2_O_2_, 5% BSA, rabbit secondary antibody, SABC (Strept Avidin–Biotin Complex), and DAB (3,3’-Diaminobenzidine) was used in conjunction with specific primary antibodies against SelK (Invitrogen, #PA5-52,529), Ki67 (Abcam, #ab16667), β-TrCP1 (Abcam, #ab233739), or CDK4 (Proteintech, #11,026–1-AP) for IHC staining according to manufacturer instructions. Each tissue section was analyzed with a single primary antibody to avoid mischaracterization of total staining intensity (due to use of the single type of secondary antibody). All stained samples were ultimately evaluated for staining intensity using a Nikon Eclipse Ni microsystem (DS-Ri2) and Image-Pro Plus (v.6.0, Media Cybernetics, Rockville, MD, USA) to capture images and to calculate the integrated optical density (IOD) of each stained area (IOD/area).

### Co-immunoprecipitation

Target plasmids were transiently transfected into cells at a 1:1 ratio. After 36 h of transfection, at which the proteins were co-expressed in the cells. The cells were then lysed on ice using cell lysis buffer (Cell Signaling Technology, #9803) supplemented with a complete protein inhibitor mixture (Roche, #04693116001). After high-speed centrifugation (13,000 × g) for 10 min, each supernatant was collected, and protein concentration was determined using a BCA kit (Thermo Fisher Scientific, #23,225). For immunoprecipitation, equal amounts of labeled magnetic beads, i.e., anti-HA-tagged mAb magnetic beads (MBL, M180-11) and anti-GFP-tagged mAb magnetic beads (MBL, D153-8), were separately incubated with an equal amount of lysate protein for 4–5 h at 4 °C. Thereafter, the beads were washed several times with cell lysis buffer to remove unbound protein. The final precipitate was then rinsed with cell lysis buffer and re-suspended in cell lysis buffer and boiled for 5 min. The supernatant from this solution was then collected using an MBL magnetic rack system and the isolated/separated protein then analyzed by Western blotting.

### Statistical analysis

Experimental data are presented as means ± SD. All data were processed and plotted with Prism software (v.6.0, GraphPad, San Diego, CA). Results from technical replicates are presented as means ± SD, with no statistical analysis performed. In contrast, results from biological replicates are also presented as means ± SD, and statistical analyses were conducted to compare differences; for each endpoint evaluated, differences between the two groups being compared at a given time were evaluated using a Student’s *t*-test. A *p*-value ≤ 0.05 was taken as statistically significant.

### Supplementary Information

Supplementary Material 1.

Supplementary Material 2.

Supplementary Material 3.

Supplementary Material 4.

Supplementary Material 5.

Supplementary Material 6.

Supplementary Material 7.

Supplementary Material 8.

## Data Availability

All data created and analyzed during this current work are involved in this published article (and its supplementary information files).
