# Supplementary material for: SelK promotes glioblastoma cell proliferation by inhibiting β-TrCP1 mediated ubiquitin-dependent degradation of CDK4
Source: J Exp Clin Cancer Res. 2024 Aug 19;43:231. doi: 10.1186/s13046-024-03157-x (PMC11331741; doi:10.1186/s13046-024-03157-x)
Supplement: Supplementary file 1 — Supplementary Material 1. [file 13046_2024_3157_MOESM1_ESM.docx]

**Supplemental Table 1** Information about the GB patient who underwent TMT-tagged quantitative proteomic analysis.

| Case | Gender | Age | Survival time（month） | Grade | IDH-Mutation | Radiotherapy and chemotherapy |
| --- | --- | --- | --- | --- | --- | --- |
| GB#1 | male | 36 | 6 | IV | Wildtype | Yes |
| GB#2 | male | 66 | 9 | IV | Wildtype | Yes |
| GB#3 | female | 57 | 7 | IV | Wildtype | Yes |
| GB#4 | female | 58 | 10.5 | IV | Wildtype | Yes |
| GB#5 | female | 63 | 11 | IV | Wildtype | Yes |
| GB#6 | male | 36 | 41 | IV | Wildtype | Yes |
| GB#7 | male | 60 | 36 | IV | Wildtype | Yes |
| GB#8 | male | 39 | 40 | IV | Wildtype | Yes |
| GB#9 | female | 54 | 46 | IV | Wildtype | Yes |
| GB#10 | female | 29 | 43 | IV | Wildtype | Yes |
