# Supplementary material for: SelK promotes glioblastoma cell proliferation by inhibiting β-TrCP1 mediated ubiquitin-dependent degradation of CDK4
Source: J Exp Clin Cancer Res. 2024 Aug 19;43:231. doi: 10.1186/s13046-024-03157-x (PMC11331741; doi:10.1186/s13046-024-03157-x)
Supplement: Supplementary file 2 — Supplementary Material 2. [file 13046_2024_3157_MOESM2_ESM.docx]

**Supplemental Table 2** Information about the GB patients including case number, grade and survival time.

| Case | Grade | Survival time （month） | Case | Grade | Survival time （month） |
| --- | --- | --- | --- | --- | --- |
| GB#1 | IV | 42 | GB#45 | IV | 16 |
| GB#2 | IV | 12 | GB#46 | IV | 14 |
| GB#3 | IV | 6.5 | GB#47 | IV | 16 |
| GB#4 | IV | 17.1 | GB#48 | IV | 10.4 |
| GB#5 | IV | 19.1 | GB#49 | IV | 33.6 |
| GB#6 | IV | 19.9 | GB#50 | IV | 21.5 |
| GB#7 | IV | 26.9 | GB#51 | IV | 17.4 |
| GB#8 | IV | 43.6 | GB#52 | IV | 8 |
| GB#9 | IV | 9 | GB#53 | IV | 8.3 |
| GB#10 | IV | 24.6 | GB#54 | IV | 1.3 |
| GB#11 | IV | 3.5 | GB#55 | IV | 2.1 |
| GB#12^*^ | IV | 46 | GB#56 | IV | 2.2 |
| GB#13 | IV | 4.5 | GB#57 | IV | 3.1 |
| GB#14 | IV | 4.2 | GB#58 | IV | 3.3 |
| GB#15 | IV | 23 | GB#59 | IV | 4.2 |
| GB#16 | IV | 5.7 | GB#60 | IV | 6.1 |
| GB#17 | IV | 55 | GB#61 | IV | 6.4 |
| GB#18 | IV | 12.5 | GB#62 | IV | 7.2 |
| GB#19 | IV | 40.1 | GB#63 | IV | 8.2 |
| GB#20 | IV | 40.1 | GB#64 | IV | 8.5 |
| GB#21 | IV | 36.2 | GB#65 | IV | 9 |
| GB#22 | IV | 38 | GB#66 | IV | 9.1 |
| GB#23 | IV | 8.9 | GB#67 | IV | 9.6 |
| GB#24 | IV | 11 | GB#68 | IV | 10.5 |
| GB#25 | IV | 4.5 | GB#69 | IV | 10.9 |
| GB#26 | IV | 7 | GB#70 | IV | 12.1 |
| GB#27 | IV | 17.4 | GB#71 | IV | 12.7 |
| GB#28 | IV | 25.1 | GB#72 | IV | 13.3 |
| GB#29 | IV | 13.3 | GB#73 | IV | 17.5 |
| GB#30 | IV | 11 | GB#74 | IV | 20.9 |
| GB#31 | IV | 16.1 | GB#75 | IV | 22 |
| GB#32 | IV | 7.2 | GB#76 | IV | 25.9 |
| GB#33 | IV | 13.6 | GB#77 | IV | 29.2 |
| GB#34 | IV | 2.6 | GB#78 | IV | 31.4 |
| GB#35 | IV | 4 | GB#79 | IV | 32.4 |
| GB#36 | IV | 27.4 | GB#80 | IV | 41.8 |
| GB#37 | IV | 7 | GB#81 | IV | 43.9 |
| GB#38 | IV | 13.4 | GB#82 | IV | 59.4 |
| GB#39 | IV | 16.8 | GB#83 | IV | 131.9 |
| GB#40 | IV | 14.1 | GB#84 | IV | 177.1 |
| GB#41 | IV | 23.5 | GB#85 | IV | 152.8 |
| GB#42 | IV | 12.4 | GB#86 | IV | 149.6 |
| GB#43 | IV | 11.5 | GB#87 | IV | 30.1 |
| GB#44 | IV | 10 | GB#88 | IV | 27.4 |

* Same patient as GB#9 in supplemental table 1.
