# Supplementary material for: SelK promotes glioblastoma cell proliferation by inhibiting β-TrCP1 mediated ubiquitin-dependent degradation of CDK4
Source: J Exp Clin Cancer Res. 2024 Aug 19;43:231. doi: 10.1186/s13046-024-03157-x (PMC11331741; doi:10.1186/s13046-024-03157-x)
Supplement: Supplementary file 3 — Supplementary Material 3. [file 13046_2024_3157_MOESM3_ESM.docx]

**Supplemental Table 3** Primer sequences for real-time fluorescence quantitative PCR.

| Primer name | primer sequence |
| --- | --- |
| qCDK4-F | 5’-CTACAGCTACCAGATGGCACTTAC-3’ |
| qCDK4-R | 5’-CAAAGATACAGCCAACACTCCACA-3’ |
| qβ-TrCP1-F | 5’-CCTTCGCTGCGATGCCTGTA-3’ |
| qβ-TrCP1-R | 5’-GGCACAGCTGTTGTATGTCTGT-3’ |
| qSKP2-F | 5’- GTGGTATCGCCTAGCGTCTG-3’ |
| qSKP2-R | 5’-GAGACAGTATGCCGTGGAGG-3’ |
| qGAPDH-F | 5’-ATCAATGGAAATCCCATCACCA-3’ |
| qGAPDH-R | 5’-GACTCCACGACGTACTCAGCG-3’ |
