# Supplementary material for: SelK promotes glioblastoma cell proliferation by inhibiting β-TrCP1 mediated ubiquitin-dependent degradation of CDK4
Source: J Exp Clin Cancer Res. 2024 Aug 19;43:231. doi: 10.1186/s13046-024-03157-x (PMC11331741; doi:10.1186/s13046-024-03157-x)
Supplement: Supplementary file 4 — Supplementary Material 4. [file 13046_2024_3157_MOESM4_ESM.pdf]

Supplementary Figure S1. Assays to examine the proliferation of U251 cells following CDK4 overexpression.

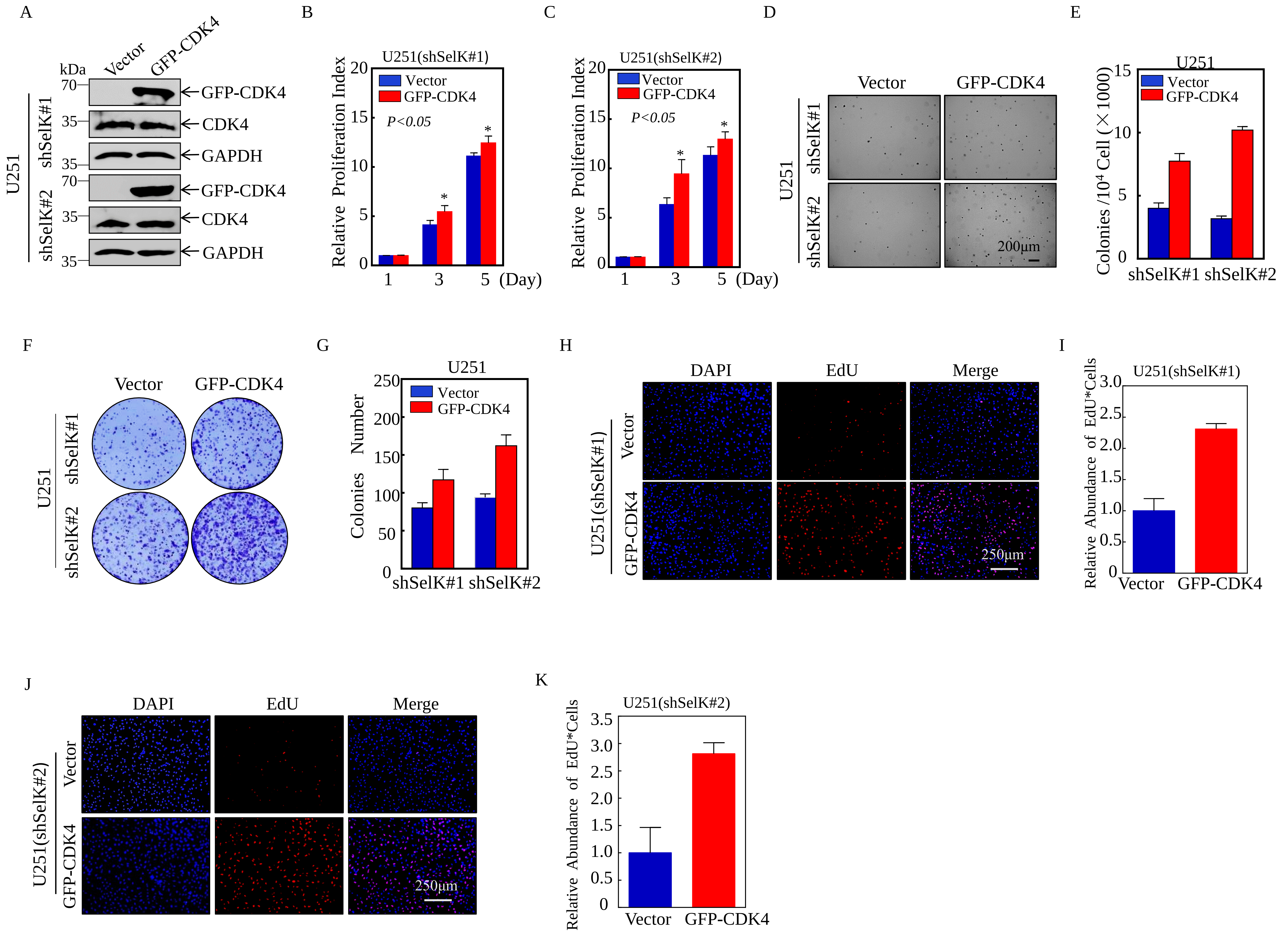

Figure S1. Assays to examine the proliferation of U251 cells following CDK4 overexpression. (A) CDK4 was stably over-expressed in U251(shSelK#1) and U251(shSelK#2) cells and detected by Western blot. (**B, C)** Effect of CDK4 over-expression on proliferation of U251(shSelK#1) and U251(shSelK#2) cells using ATP assay (each assay repeated three times independently). U251(shSelK#1/CDK4) and U251 (shSelK#2/CDK4), and control cell clone formation numbers via (**D, E)** soft agar assay and **(F, G)** clonogenic assay. (**H-K)** Effect of CDK4 over-expression on DNA replication activity in U251(shSelK#1) and U251(shSelK#2) (EdU assay). *Significant difference at $p < 0.05$. All data are expressed as means ± SD.
