# Supplementary material for: SelK promotes glioblastoma cell proliferation by inhibiting β-TrCP1 mediated ubiquitin-dependent degradation of CDK4
Source: J Exp Clin Cancer Res. 2024 Aug 19;43:231. doi: 10.1186/s13046-024-03157-x (PMC11331741; doi:10.1186/s13046-024-03157-x)
Supplement: Supplementary file 5 — Supplementary Material 5. [file 13046_2024_3157_MOESM5_ESM.pdf]

Supplementary Figure S2. Assays to examine the effects of 2-APB on CDK4 degradation and proliferation

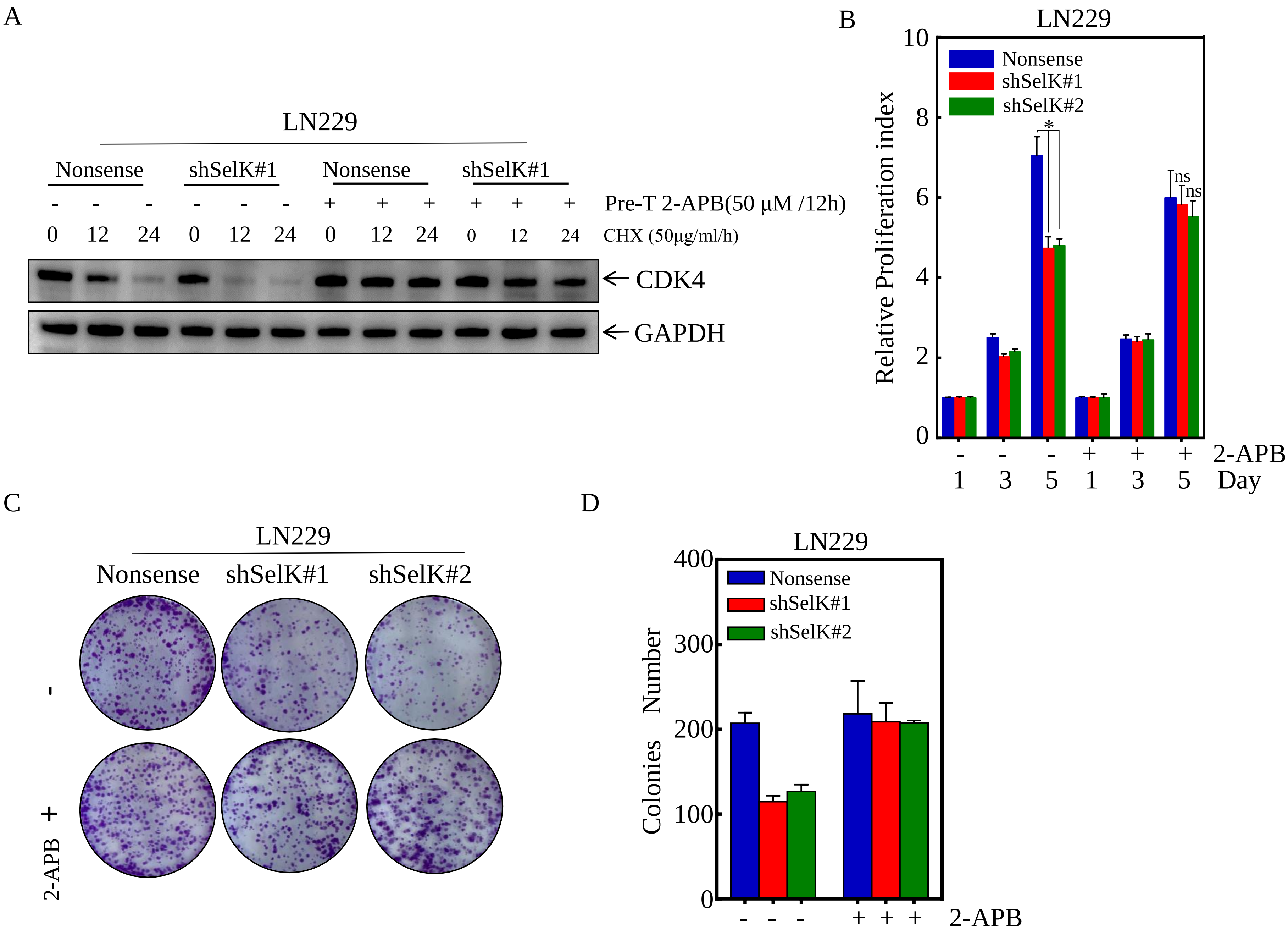

Figure S2. Assays to examine the effects of 2-APB on CDK4 degradation and proliferation. (A) The degradation rate of CDK4 was examined in LN229 (shSelK#1/Nonsense) cells by western blotting after treatment with 50 µM 2-APB for 12 h and incubation with CHX. **(B)** ATP assay to determine effect of SelK knockdown on proliferation of LN229cells after pre-treatment with 2-APB (50 µM) for 12 h,(each assay repeated three times independently). **(C,D)** Clonogenic assay to determine effect of SelK knockdown on the proliferation of LN229 after pre-treatment with 2-APB (50 µM) for 12 h. *Significant difference at $p < 0.05$. All data are expressed as means ± SD.
