# Supplementary material for: SelK promotes glioblastoma cell proliferation by inhibiting β-TrCP1 mediated ubiquitin-dependent degradation of CDK4
Source: J Exp Clin Cancer Res. 2024 Aug 19;43:231. doi: 10.1186/s13046-024-03157-x (PMC11331741; doi:10.1186/s13046-024-03157-x)
Supplement: Supplementary file 6 — Supplementary Material 6. [file 13046_2024_3157_MOESM6_ESM.pdf]

Supplementary Figure S3. CDK4, β-TrCP1 and GRP78 protein levels in subcutaneous GB tumors heterologously-expressing SelK in nude mice (IHC).

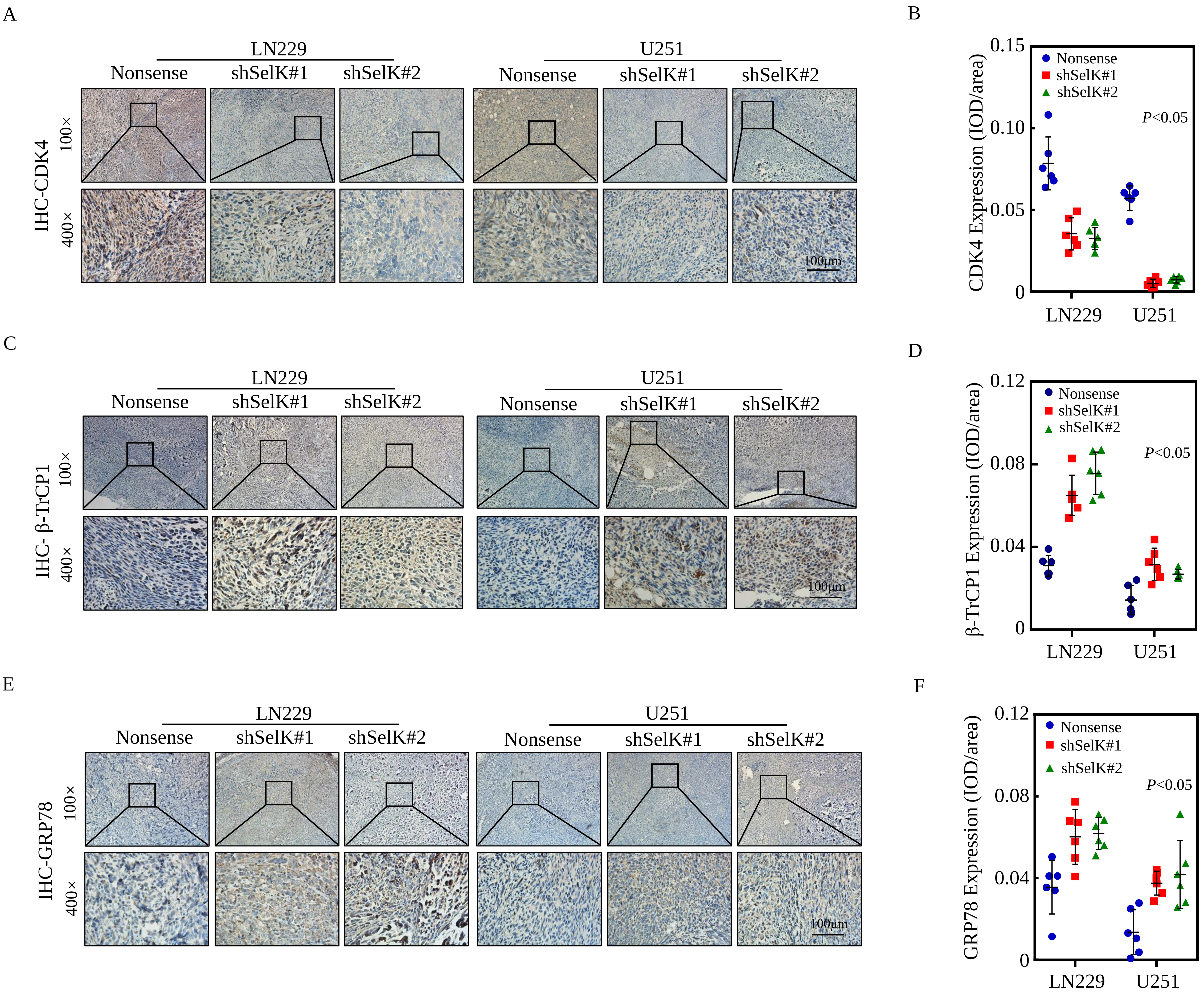

Supplementary Figure S3. (A, B) CDK4 protein levels in subcutaneous GB tumors heterologously-expressing SelK in nude mice (IHC). (**C, D)** β-TrCP1 protein levels in subcutaneous GB tumors heterologously-expressing SelK in nude mice (IHC). **(E, F)** GRP78 protein levels in subcutaneous GB tumors heterologously-expressing SelK in nude mice (IHC). *Significant difference at $p < 0.05$. All data are expressed as means ± SD.
