# Supplementary material for: SelK promotes glioblastoma cell proliferation by inhibiting β-TrCP1 mediated ubiquitin-dependent degradation of CDK4
Source: J Exp Clin Cancer Res. 2024 Aug 19;43:231. doi: 10.1186/s13046-024-03157-x (PMC11331741; doi:10.1186/s13046-024-03157-x)

Figure2

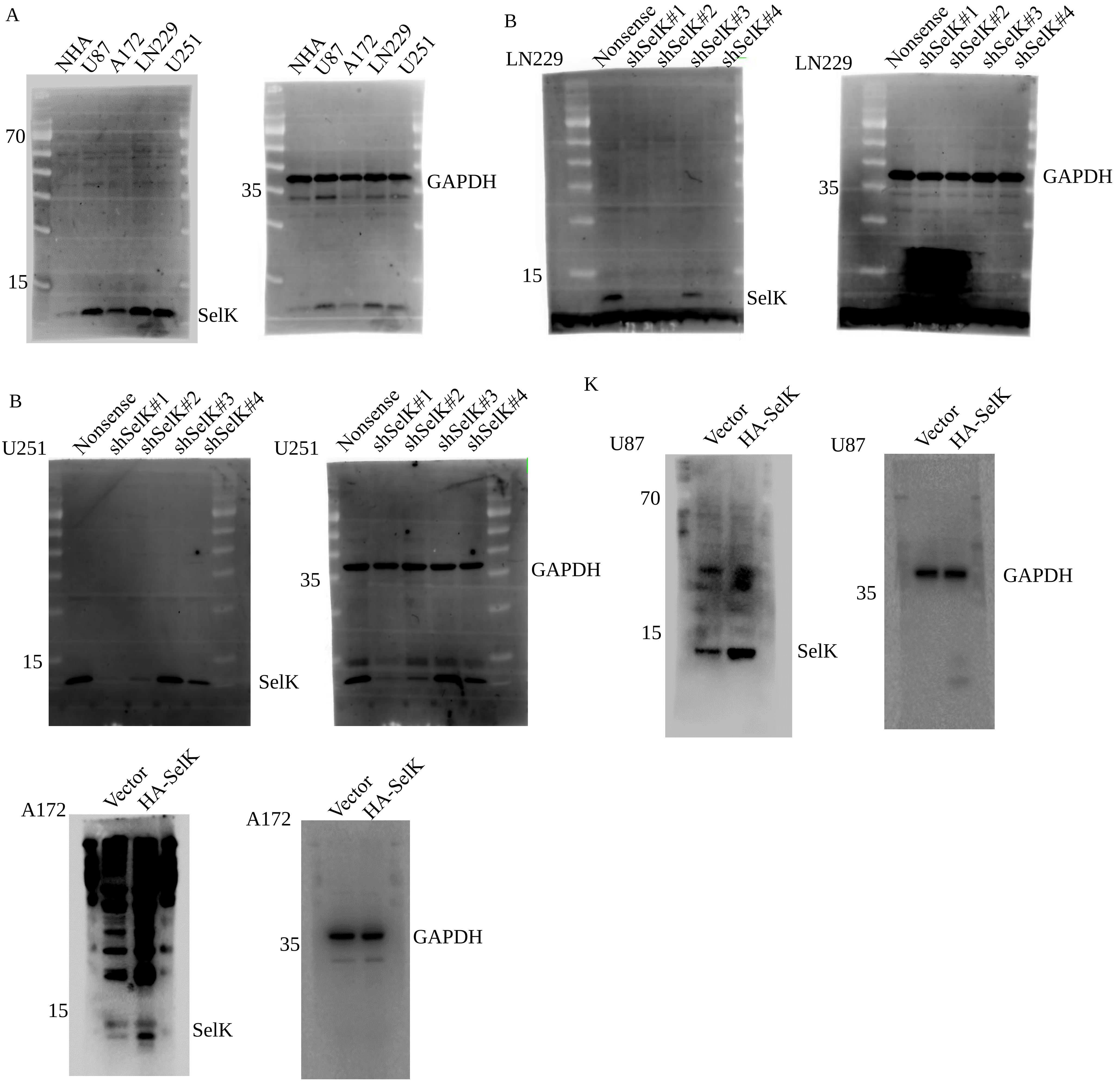
A
NHA
U87
A172
LN229
U251
70
15
SelK
35
GAPDH
B
LN229
Nonsense
shSelK#1
shSelK#2
shSelK#3
shSelK#4
15
SelK
35
GAPDH
B
U251
15
SelK
35
GAPDH
K
U87
Vector
HA-SelK
70
15
SelK
35
GAPDH
A172
15
SelK
35
GAPDH

Figure4

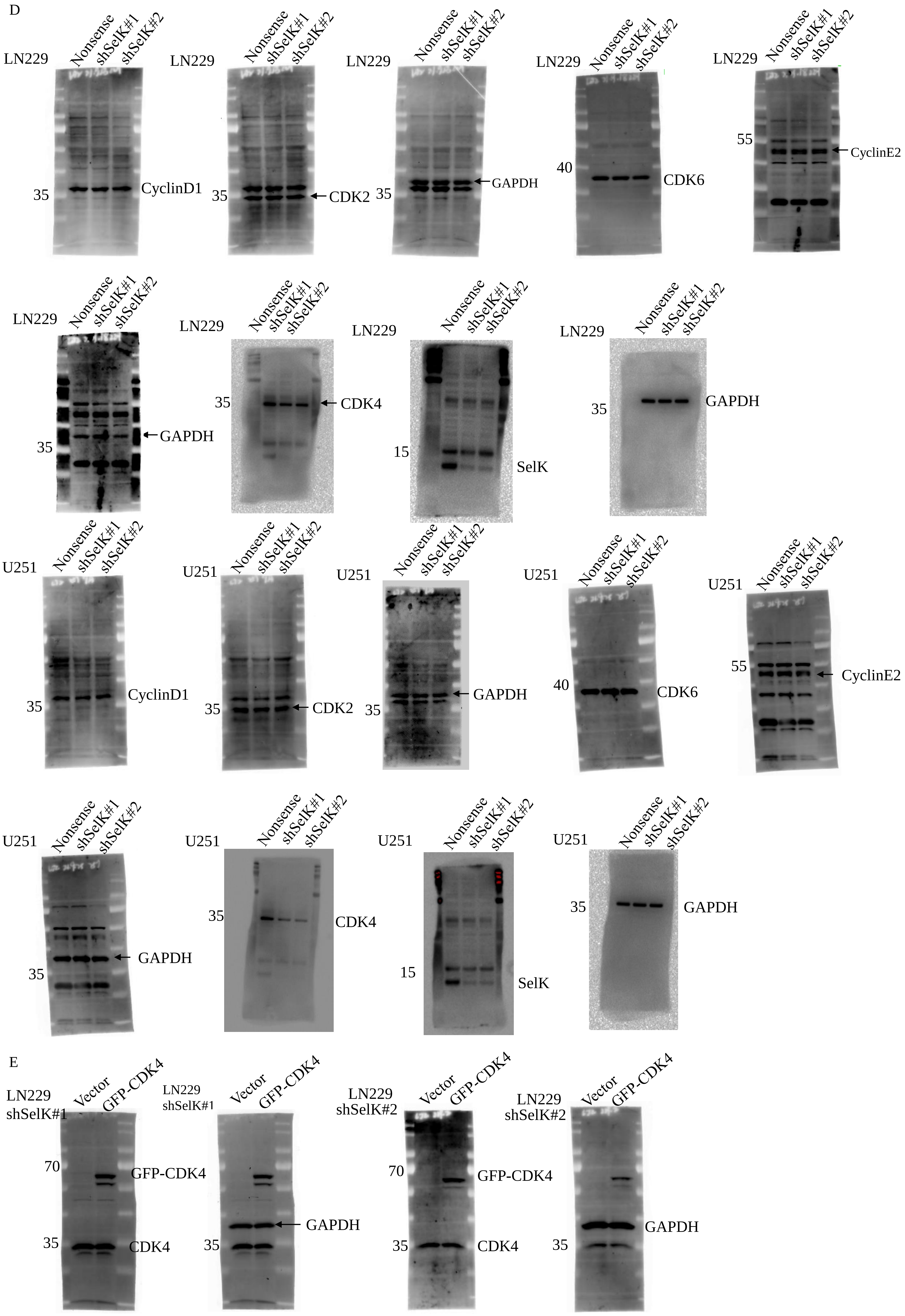
D
LN229
Nonsense
shSelK#1
shSelK#2
35
CyclinD1
CDK2
GAPDH
40
CDK6
55
CyclinE2
CDK4
15
SelK
U251
E
LN229 shSelK#1
LN229 shSelK#2
Vector
GFP-CDK4
70
GFP-CDK4
CDK4
GAPDH

Figure5

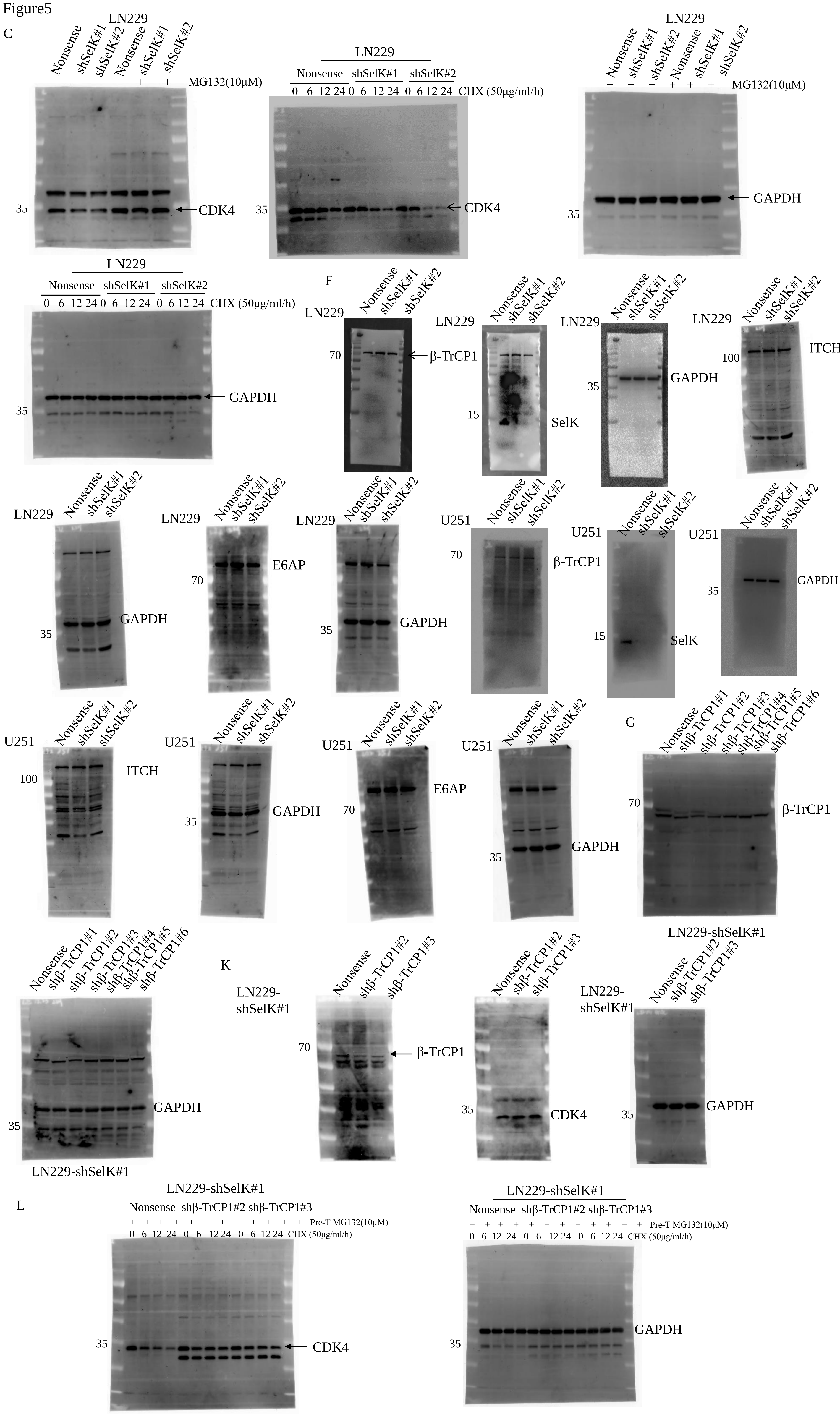

C
LN229
Nonsense shSelK#1 shSelK#2 Nonsense shSelK#1 shSelK#2
− − − + + + MG132(10μM)
35
CDK4
LN229
Nonsense shSelK#1 shSelK#2
0 6 12 24 0 6 12 24 0 6 12 24 CHX (50μg/ml/h)
35
CDK4
LN229
Nonsense shSelK#1 shSelK#2 Nonsense shSelK#1 shSelK#2
− − − + + + MG132(10μM)
GAPDH
35
LN229
Nonsense shSelK#1 shSelK#2
0 6 12 24 0 6 12 24 0 6 12 24 CHX (50μg/ml/h)
GAPDH
35
F
LN229
Nonsense shSelK#1 shSelK#2
70
β-TrCP1
LN229
15
SelK
LN229
35
GAPDH
LN229
100
ITCH
LN229
GAPDH
35
LN229
E6AP
70
LN229
35
GAPDH
U251
70
β-TrCP1
U251
15
SelK
U251
35
GAPDH
U251
100
ITCH
U251
35
GAPDH
U251
E6AP
70
U251
GAPDH
35
G
Nonsense shβ-TrCP1#1 shβ-TrCP1#2 shβ-TrCP1#3 shβ-TrCP1#4 shβ-TrCP1#5 shβ-TrCP1#6
70
β-TrCP1
LN229-shSelK#1
GAPDH
35
LN229-shSelK#1
K
LN229-shSelK#1
Nonsense shβ-TrCP1#2 shβ-TrCP1#3
70
β-TrCP1
35
CDK4
LN229-shSelK#1
35
GAPDH
L
LN229-shSelK#1
Nonsense shβ-TrCP1#2 shβ-TrCP1#3
+ + + + + + + + + + + + Pre-T MG132(10μM)
0 6 12 24 0 6 12 24 0 6 12 24 CHX (50μg/ml/h)
35
CDK4
LN229-shSelK#1
Nonsense shβ-TrCP1#2 shβ-TrCP1#3
+ + + + + + + + + + + + Pre-T MG132(10μM)
0 6 12 24 0 6 12 24 0 6 12 24 CHX (50μg/ml/h)
GAPDH
35

Figure6

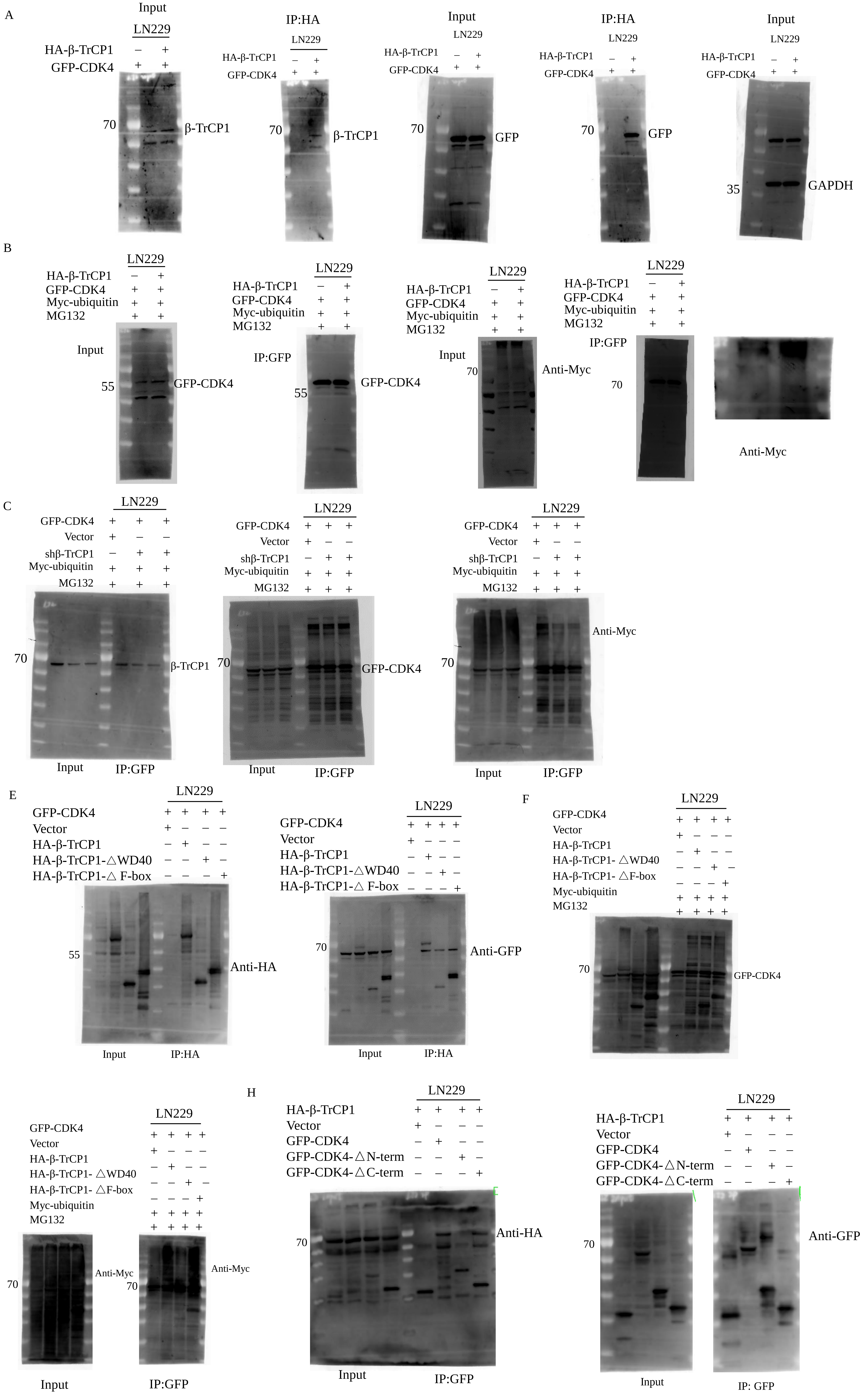

A
Input
LN229
HA-β-TrCP1 – +
GFP-CDK4 + +
70
β-TrCP1
IP:HA
LN229
HA-β-TrCP1 – +
GFP-CDK4 + +
70
β-TrCP1
Input
LN229
HA-β-TrCP1 – +
GFP-CDK4 + +
70
GFP
IP:HA
LN229
HA-β-TrCP1 – +
GFP-CDK4 + +
70
GFP
Input
LN229
HA-β-TrCP1 – +
GFP-CDK4 + +
35
GAPDH
B
LN229
HA-β-TrCP1 – +
GFP-CDK4 + +
Myc-ubiquitin + +
MG132 + +
Input
55
GFP-CDK4
IP:GFP
55
GFP-CDK4
Input
70
Anti-Myc
IP:GFP
70
Anti-Myc
C
LN229
GFP-CDK4 + + +
Vector + – –
shβ-TrCP1 – + +
Myc-ubiquitin + + +
MG132 + + +
70
β-TrCP1
Input
IP:GFP
70
GFP-CDK4
Input
IP:GFP
Anti-Myc
70
Input
IP:GFP
E
LN229
GFP-CDK4 + + + +
Vector + – – –
HA-β-TrCP1 – + – –
HA-β-TrCP1-△WD40 – – + –
HA-β-TrCP1-△ F-box – – – +
55
Anti-HA
Input
IP:HA
70
Anti-GFP
Input
IP:HA
F
LN229
GFP-CDK4 + + + +
Vector + – – –
HA-β-TrCP1 – + – –
HA-β-TrCP1- △WD40 – – + –
HA-β-TrCP1- △F-box – – – +
Myc-ubiquitin + + + +
MG132 + + + +
70
GFP-CDK4
70
Anti-Myc
Input
70
Anti-Myc
IP:GFP
H
LN229
HA-β-TrCP1 + + + +
Vector + – – –
GFP-CDK4 – + – –
GFP-CDK4-△N-term – – + –
GFP-CDK4-△C-term – – – +
70
Anti-HA
Input
IP:GFP
70
Anti-GFP
Input
IP: GFP

Figure7

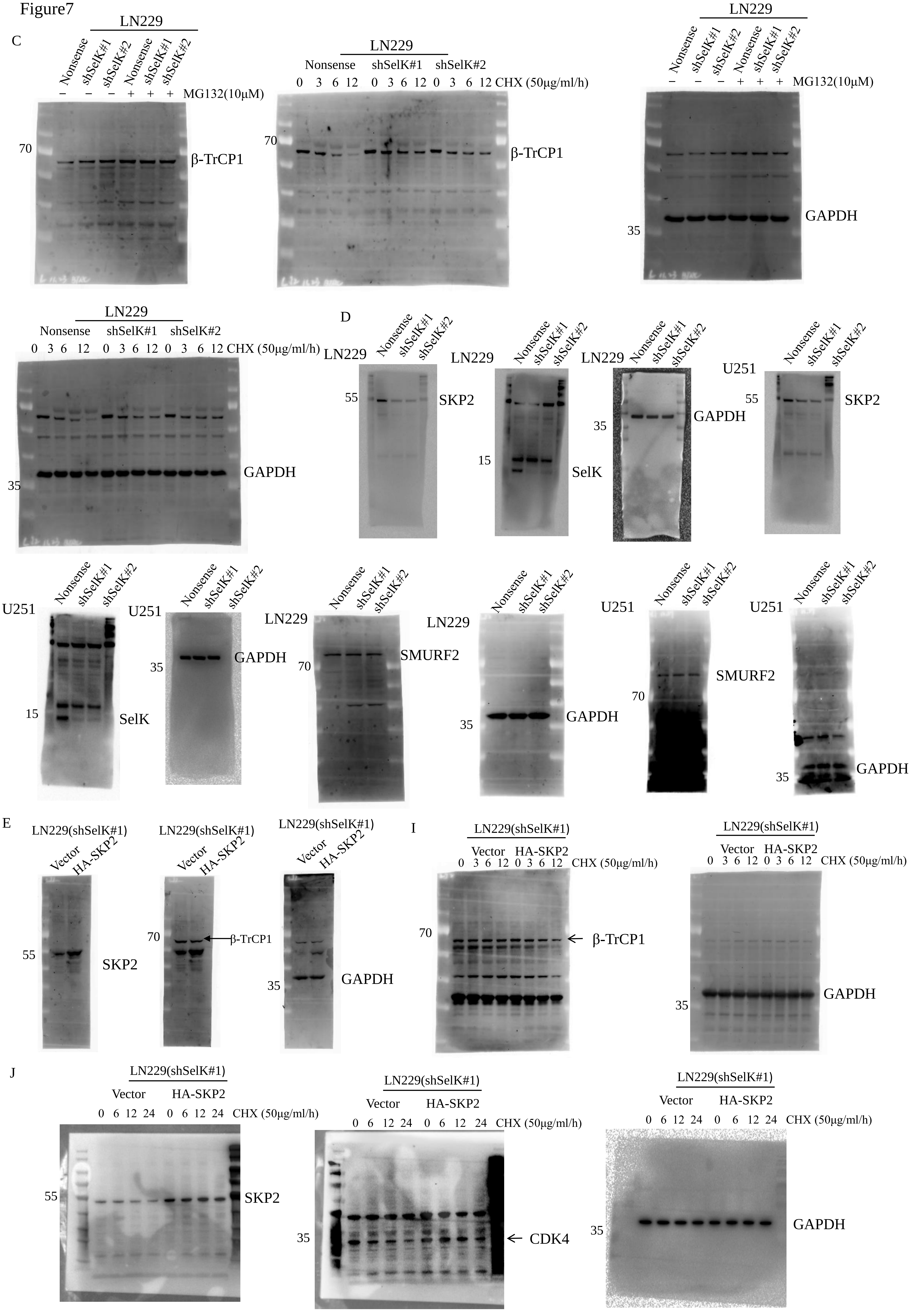
C
LN229
Nonsense shSelK#1 shSelK#2 Nonsense shSelK#1 shSelK#2
− − − + + + MG132(10μM)
70
β-TrCP1
LN229
Nonsense shSelK#1 shSelK#2
0 3 6 12 0 3 6 12 0 3 6 12 CHX (50μg/ml/h)
70
β-TrCP1
LN229
Nonsense shSelK#1 shSelK#2 Nonsense shSelK#1 shSelK#2
− − − + + + MG132(10μM)
GAPDH
35
LN229
Nonsense shSelK#1 shSelK#2
0 3 6 12 0 3 6 12 0 3 6 12 CHX (50μg/ml/h)
GAPDH
35
D
LN229
Nonsense shSelK#1 shSelK#2
55
SKP2
LN229
Nonsense shSelK#1 shSelK#2
15
SelK
LN229
Nonsense shSelK#1 shSelK#2
35
GAPDH
U251
Nonsense shSelK#1 shSelK#2
55
SKP2
U251
Nonsense shSelK#1 shSelK#2
15
SelK
U251
Nonsense shSelK#1 shSelK#2
35
GAPDH
LN229
Nonsense shSelK#1 shSelK#2
70
SMURF2
LN229
Nonsense shSelK#1 shSelK#2
35
GAPDH
U251
Nonsense shSelK#1 shSelK#2
70
SMURF2
U251
Nonsense shSelK#1 shSelK#2
GAPDH
35
E
LN229(shSelK#1)
Vector HA-SKP2
55
SKP2
LN229(shSelK#1)
Vector HA-SKP2
70
β-TrCP1
LN229(shSelK#1)
Vector HA-SKP2
35
GAPDH
I
LN229(shSelK#1)
Vector HA-SKP2
0 3 6 12 0 3 6 12 CHX (50μg/ml/h)
70
β-TrCP1
LN229(shSelK#1)
Vector HA-SKP2
0 3 6 12 0 3 6 12 CHX (50μg/ml/h)
GAPDH
35
J
LN229(shSelK#1)
Vector HA-SKP2
0 6 12 24 0 6 12 24 CHX (50μg/ml/h)
55
SKP2
LN229(shSelK#1)
Vector HA-SKP2
0 6 12 24 0 6 12 24 CHX (50μg/ml/h)
35
CDK4
LN229(shSelK#1)
Vector HA-SKP2
0 6 12 24 0 6 12 24 CHX (50μg/ml/h)
35
GAPDH

Figure8

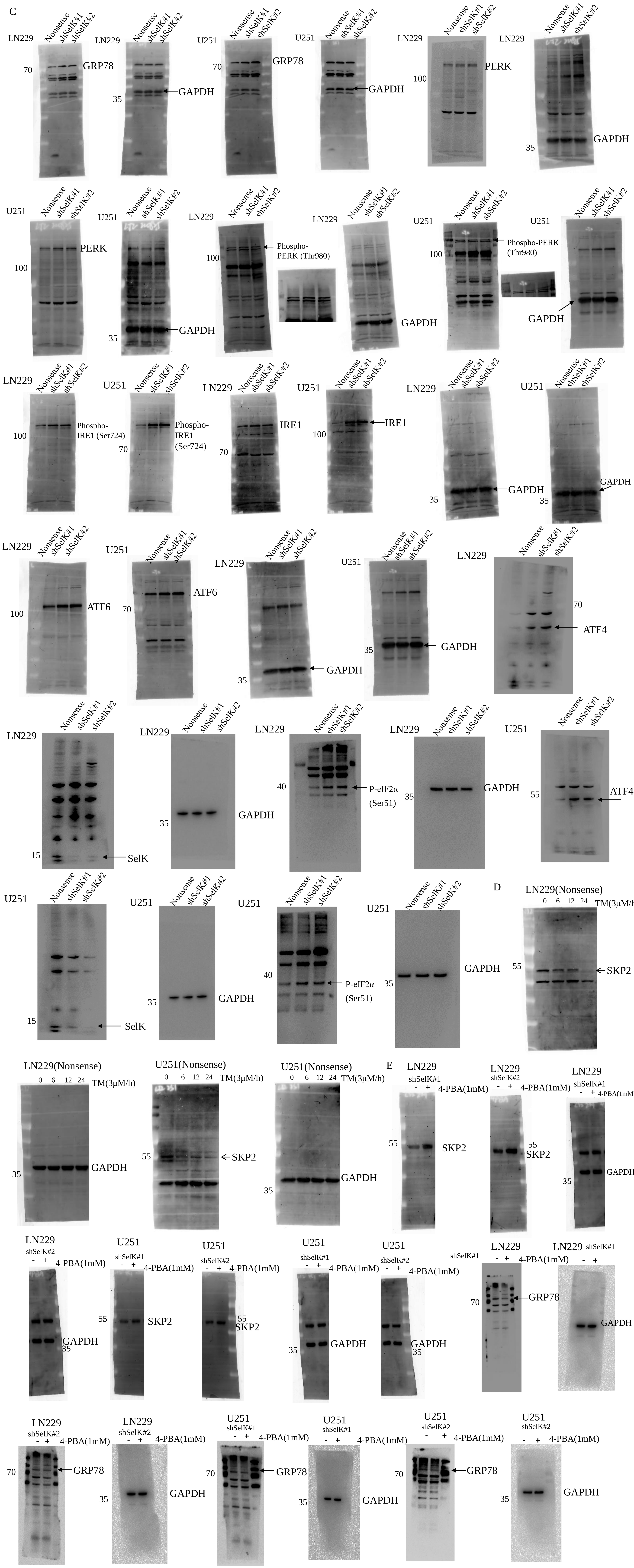
C
LN229
U251
Nonsense
shSelK#1
shSelK#2
GRP78
GAPDH
PERK
Phospho-PERK (Thr980)
Phospho-IRE1 (Ser724)
IRE1
ATF6
ATF4
SelK
P-eIF2α (Ser51)
100
70
55
40
35
15
D
LN229(Nonsense)
U251(Nonsense)
0
6
12
24
TM(3μM/h)
SKP2
E
4-PBA(1mM)
-
+

Supplementary Figure S1

A

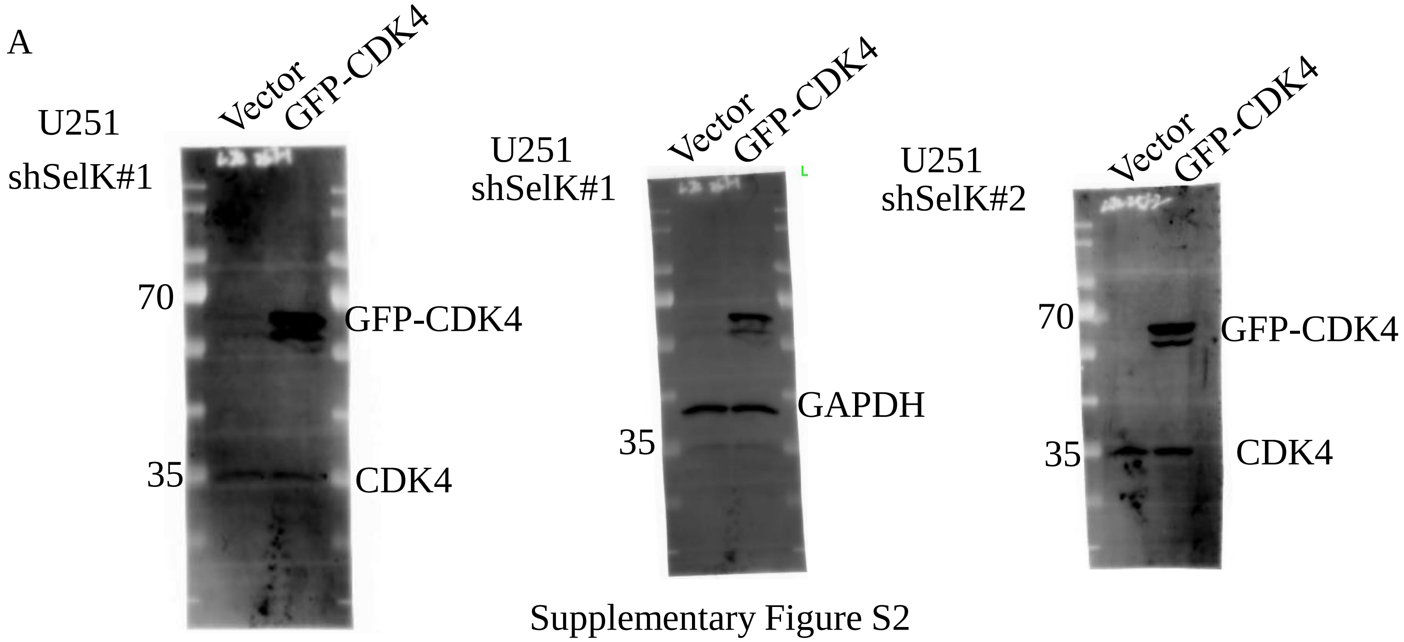

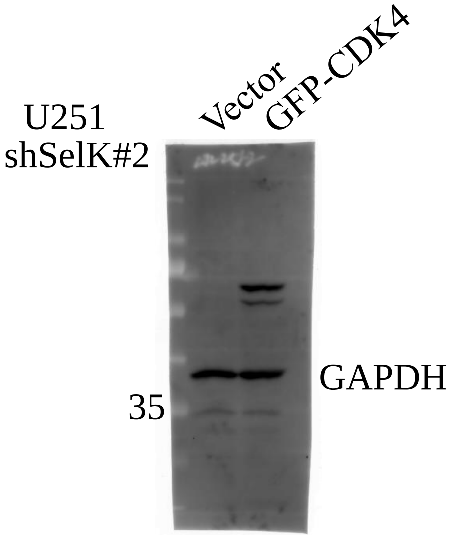

Supplementary Figure S2

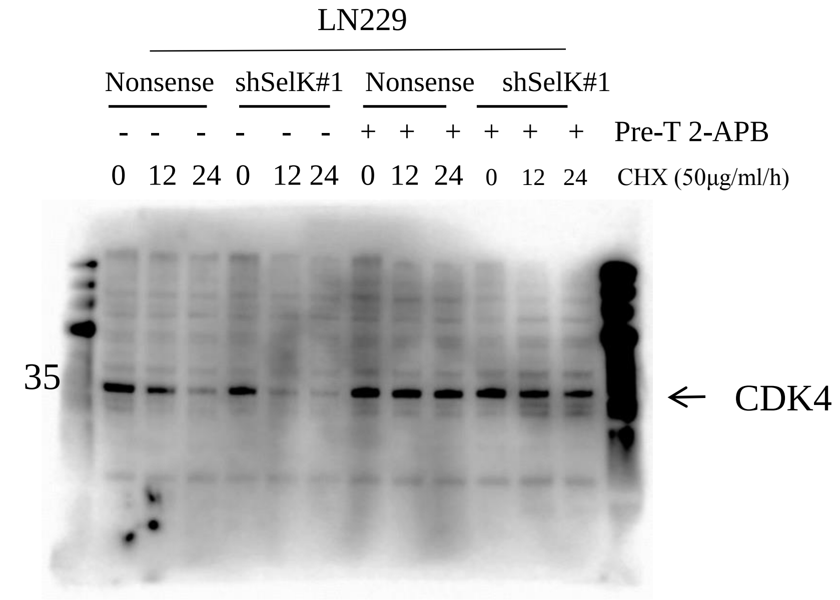

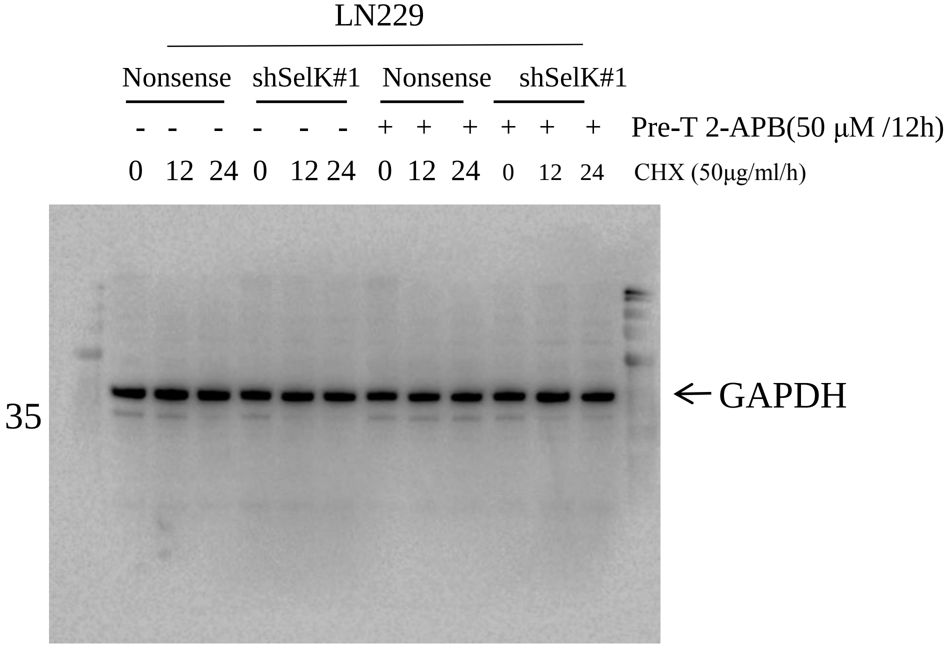

Supplement: Supplementary file 8 — Supplementary Material 8. [file 13046_2024_3157_MOESM8_ESM.pdf]
